# A novel DNA methylation signature associated with lymph node metastasis status in early gastric cancer

**DOI:** 10.1186/s13148-021-01219-x

**Published:** 2022-02-03

**Authors:** Shang Chen, Yanqi Yu, Tao Li, Weimei Ruan, Jun Wang, Quanzhou Peng, Yingdian Yu, Tianfeng Cao, Wenyuan Xue, Xin Liu, Zhiwei Chen, Jiang Yu, Jian-Bing Fan

**Affiliations:** 1 Department of Biochemistry and Molecular Biology, School of Basic Medical Sciences, Southern Medical University, Guangzhou, 510515 China; 2 Department of Pathology, School of Basic Medical Sciences, Southern Medical University, Guangzhou, 510515 China; 3 Department of General Surgery, Nanfang Hospital, Southern Medical University, Guangzhou, 510515 China; 4 AnchorDx Medical Co., Ltd, Unit 502, No. 8, 3rd Luoxuan Road, International Bio-Island, Guangzhou, 510300 China; 5 Department of Pathology, Shenzhen People’s Hospital, Shennan Dong Lu, Luohu District, Shenzhen, 518002 China; 6 AnchorDx, Inc., 46305 Landing Pkwy, Fremont, CA 94538 USA

**Keywords:** Early gastric cancer, Methylation markers, Lymph node metastasis, Early detection

## Abstract

**Background:**

Lymph node metastasis (LNM) is an important factor for both treatment and prognosis of early gastric cancer (EGC). Current methods are insufficient to evaluate LNM in EGC due to suboptimal accuracy. Herein, we aim to identify methylation signatures for LNM of EGC, facilitate precision diagnosis, and guide treatment modalities.

**Methods:**

For marker discovery, genome-wide methylation sequencing was performed in a cohort (marker discovery) using 47 fresh frozen (FF) tissue samples. The identified signatures were subsequently characterized for model development using formalin-fixed paraffin-embedded (FFPE) samples by qPCR assay in a second cohort (model development cohort, *n* = 302, training set: *n* = 151, test set: *n* = 151). The performance of the established model was further validated using FFPE samples in a third cohorts (validation cohort, *n* = 130) and compared with image-based diagnostics, conventional clinicopathology-based model (conventional model), and current standard workups.

**Results:**

Fifty LNM-specific methylation signatures were identified de novo and technically validated. A derived 3-marker methylation model for LNM diagnosis was established that achieved an AUC of 0.87 and 0.88, corresponding to the specificity of 80.9% and 85.7%, sensitivity of 80.6% and 78.1%, and accuracy of 80.8% and 83.8% in the test set of model development cohort and validation cohort, respectively. Notably, this methylation model outperformed computed tomography (CT)-based imaging with a superior AUC (0.88 vs. 0.57, *p* < 0.0001) and individual clinicopathological features in the validation cohort. The model integrated with clinicopathological features demonstrated further enhanced AUCs of 0.89 in the same cohort. The 3-marker methylation model and integrated model reduced 39.4% and 41.5% overtreatment as compared to standard workups, respectively.

**Conclusions:**

A novel 3-marker methylation model was established and validated that shows diagnostic potential to identify LNM in EGC patients and thus reduce unnecessary gastrectomy in EGC.

**Supplementary Information:**

The online version contains supplementary material available at 10.1186/s13148-021-01219-x.

## Background

Early gastric cancer (EGC), with an invasion depth limited to mucosa or submucosa, accounts for approximately 10–20% of gastric cancer [1, 2]. Lymph node metastasis (LNM) status is one of the most important clinical factors affecting the prognosis of gastric cancer; the incidence of LNM in EGC is about 8–25% [3, 4]. Endoscopic submucosal dissection (ESD) and endoscopic mucosal resection (EMR) are the mainstream approaches for LNM treatment in low-risk EGC patients, due to the minimally invasive, function-preserving, en bloc resection, limited trauma, and maintenance of a good quality of life [5, 6]. However, for EGC patients at high risk of LNM, radical gastrectomy with a lymphadenectomy is usually adopted. However, it could lead to various post-gastrectomy complications that include anastomotic leakage, bleeding, stricture, delayed gastric emptying, reflux esophagitis, residual food, and reduced quality of life postoperatively [5, 6]. Therefore, precise assessment of lymph node metastatic status in EGC plays a critical role in the treatment decision making.

Currently, LNM is diagnosed mainly by imaging methods, such as endoscopic ultrasonography, computed tomography (CT), positron emission tomography with CT (PET-CT), or by evaluating clinicopathological features after endoscopic biopsy, including submucosal invasion, ulceration, undifferentiated type, and lymphovascular invasion status [7–9]. However, the accuracy and reliability of these methods are unsatisfactory, leading to overtreatment and unnecessary gastrectomy in a large portion of EGC patients [10–12]. Post-gastrectomy pathological evaluation showed that about 80% of EGC patients with negative lymph node metastasis were treated unnecessarily with radical gastrectomy [10, 11]. This suggests that the current standard of care in the clinical setting for LNM diagnosis is inadequate and it is imperative to develop novel methods to accurately determine LNM status and improve the quality of life in patients with EGC.

DNA methylation is one of the most important epigenetic modifications. A growing number of studies have shown that DNA methylation plays a prominent role in tumorigenesis and progression [13, 14]. Abnormal DNA methylation occurs before the clinical symptoms of the disease become apparent and often leads to gene misexpression [15]. With the development of high-throughput technologies, cancerous genome-wide methylation data have been used to study potential markers of early diagnosis, prognostic assessment, progression monitoring, and chemoradiotherapy sensitivity [16]. To accurately assess the possibility of LNM in EGC, numerous studies have reported different prediction models, which are constructed mainly based on clinicopathological features [17, 18]. To our knowledge, genome-wide DNA methylation mapping and modeling prediction using methylation markers for LNM in EGC have not yet been reported.

Our previous studies have shown that a genome-wide DNA methylation approach can be applied to the diagnosis of bladder cancer and the identification of benign and malignant pulmonary nodules [19, 20]. In this study, we performed a DNA methylation profiling of LNM in EGC patients and developed a methylation test for LNM diagnosis.

## Methods and materials

### Study design and patient recruitment

**Fig. 1:**
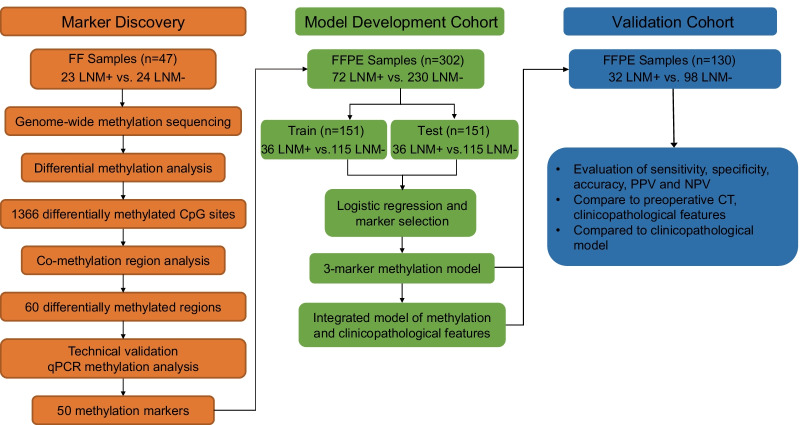
Schematic workflow of the study design

A three-phase strategy was designed in our study (Fig. 1) which included a marker discovery cohort (*n* = 47, fresh frozen (FF) tissue samples), a model development cohort (*n* = 302, formalin-fixed paraffin-embedded (FFPE) samples), and a validation cohort (*n* = 130, FFPE samples). The genome-wide methylation sequencing was applied using FF samples to identify LNM-specific methylation markers which were subsequently validated by a qPCR assay. The identified and validated methylation markers were further characterized in the model development cohort using FFPE samples as the same sample type in a practical clinical setting. The diagnostic model developed was further validated and compared to imaging diagnostics, clinicopathology-based model (conventional model), and current standard workups in the validation cohorts. An overview of the patient recruitment workflow is described in Additional file 1: Figure S1. Patients with treatment-naïve EGC were enrolled from Nanfang Hospital (*n* = 436, 47 fresh frozen FF samples and 389 FFPE samples) and Shenzhen People's Hospital (*n* = 189, FFPE samples) between January 2015 and November 2020. Samples with failed experimental QCs (*n* = 146) were excluded from the study. The tissue samples from the EGC patients were surgical specimens and collected before radiation or chemotherapy. The tumor content over 30% of the FFPE samples was confirmed by pathologists. The pathology and LNM status of the samples were confirmed by at least two gastrointestinal pathologists. The clinicopathological characteristics of all patients inducing gender, age, tumor size, tumor location, differentiation, invasional depth, ulceration, and lymphovascular invasion (LVI) are summarized in Table 1.

**Table 1:** Characteristics of EGC patients in the model development and validation cohorts

Characteristics	Model development cohort		Validation cohort
	Training set, *n* = 151, (%)	Test set, *n* = 151, (%)	Validation set, *n* = 130, (%)
*Age (years)*			
< 60	85 (56.3)	78 (51.7)	76 (58.5)
≥ 60	66 (43.7)	73 (48.3)	54 (41.5)
*Gender*			
Male	88 (58.3)	93 (61.6)	81 (62.3)
Female	63 (41.7)	58 (38.4)	49 (37.7)
*Tumor location*			
Upper	18 (11.9)	21 (13.9)	21 (16.2)
Middle	21 (13.9)	29 (19.2)	26 (20.0)
Low	112 (74.2)	101 (66.9)	83 (63.8)
*Tumor size (mm)*			
≤ 20	86 (56.9)	91 (60.3)	67 (51.5)
> 20	65 (43.1)	60 (39.7)	63 (48.5)
*Differentiation*			
Differentiated	40 (26.5)	51 (33.8)	43 (33.1)
Undifferentiated	111 (73.5)	100 (66.2)	87 (66.9)
*Invasional depth*			
Mucosa	67 (44.4)	77 (51.0)	46 (35.4)
Submucosa	84 (55.6)	74 (49.0)	84 (64.6)
*LVI*			
Presence	19 (12.6)	19 (12.6)	18 (13.8)
Absence	132 (87.4)	132 (87.4)	112 (86.2)
*Ulceration*			
Presence	52 (34.4)	56 (37.1)	36 (27.7)
Absence	99 (65.6)	95 (62.9)	94 (72.3)
*LNM*			
LNM+	36 (23.8)	36 (23.8)	32 (24.6)
LNM−	115 (76.2)	115 (76.2)	98 (75.4)

LNM, lymph node metastasis; LNM+, samples of EGC patients with positive lymph node metastasis; LNM−, samples of EGC patients without lymph node metastasis; and LVI, lymphovascular invasion

### Discovery of differentiated methylation markers

To identify potential markers, we gathered 47 FF samples of EGC. There were 23 cases of LNM+ tumor and 24 cases of LNM− tumor. Sample genomic DNA was individually constructed genome-wide methylation library using TruSeq® Methyl Capture EPIC Library Prep Kit (Illumina, USA, Catalog No. FC-151-1002) following the instructions; we refer to the latter as EPIC. The detailed patient clinicopathological features in EPIC genome-wide methylation libraries are shown in Additional file 2: Table S1. After EPIC libraries were tested by Agilent High Sensitivity DNA Kit (Agilent, USA, Catalog No. 5067-4626) for quality assurance, high-throughput sequencing was performed on Illumina's X-Ten platform. The sequencing data processing methods are detailed in Additional file 2: Methods.

### DNA extraction, bisulfite treatment, and methylation analysis by qPCR

Genomic DNAs were extracted from the FF specimens and FFPE tissue samples using the AllPrep DNA/RNA Mini Kit (Qiagen, Germany, Catalog No. 80204) and AllPrep DNA/RNA FFPE Kit (Qiagen, Germany, Catalog No. 80234) following the manufacturer’s instruction, respectively. Both genomic DNAs were quantified by the Qubit dsDNA HS Assay Kit (Thermo Fisher Scientific, USA, Catalog No. Q32851). The quality control criteria of the EGC samples required that the DNA amount was greater than 100 ng and the main bands from the agarose gel electrophoresis were above 500 bp. Bisulfite treatment was implemented using 50 ng of genomic DNA of each FFPE tissue sample with the EZ-96-DNA Methylation-Direct MagPrep Kit (Zymo Research, USA, Catalog No. D5044) according to the manufacturer’s recommendations. Subsequently, a 50-marker EGC-LNM DNA methylation panel (Additional file 2: Table S2) was designed and used to characterize the methylation patterns in EGC-LNM patients with the EGC-LNM detection kit (AnchorDx, China, Catalog No. EGME-002). The methylation analysis by MethyLight approach was described earlier (details are in Additional file 2: Methods) [21]. The qPCR methylation analysis was performed on the Quant Studio 3 Real-Time PCR System (Thermo Fisher, USA). Then, the diagnostic model of LNM in EGC was established and validated based on methylation-specific qPCR data.

### Methylation model development and validation

432 FFPE samples were randomly divided into modeling development cohort (*n* = 302) and validation cohort (*n* = 130) at a ratio of approximately 7:3. The cohort division was blinded to the methylation test results. The model development cohort (*n* = 302) was further randomly split into 50% training and 50% testing sets with a 20-fold validation. The identified 50 markers were analyzed with the least absolute shrinkage and selection operator (LASSO) algorithm to determine the minimum marker requirement and select top markers. The selected top markers were further used for model construction with logistic regression algorithm by iterative marker combination analysis in the model development cohort. A validation (*n* = 130) cohort was used to independently test the final model. Sensitivity, specificity, accuracy, positive predictive value, and negative predictive value were then evaluated.

### Development and evaluation of the conventional model and integrated model

The 8 clinicopathological variables were included in the univariate analysis to explore the association with LNM in the model development cohort, and variables with a *p* value less than 0.05 were included in multivariate analysis for the conventional model. Forward stepwise regression analysis evaluated odds ratio (OR) values with a 95% CI to identify independent predictors. The integrated model was built according to independent predictors and the 3-gene methylation signature. Tolerance and variation inflation factors were used to evaluate the multicollinearity of multivariate models. Based on both multivariate logistic regression models, two quantitative scoring formulas were derived and the area under the receiver operating characteristic curve (AUROC) was measured. (Details are in Additional file 2: Methods.)

### Statistical analysis

Wilcoxon signed-rank test or Mann–Whitney *U* test were used to analyze epigenome methylation data. Student *t* test was used to evaluate the distribution of risk scores among different test groups. The *χ*^2^ test or Fisher's exact test and two-tailed *t* test were used to compare categorical and continuous variables, when appropriate. Logistic regression-based model constructions were conducted using R glmnet (2.0.16) packages. Other details of the statistical analyses are described in Additional file 2: Methods.

## Results

### Genome-wide screening of DNA methylation markers to detect LNM in EGC tissue samples

**Fig. 2:**
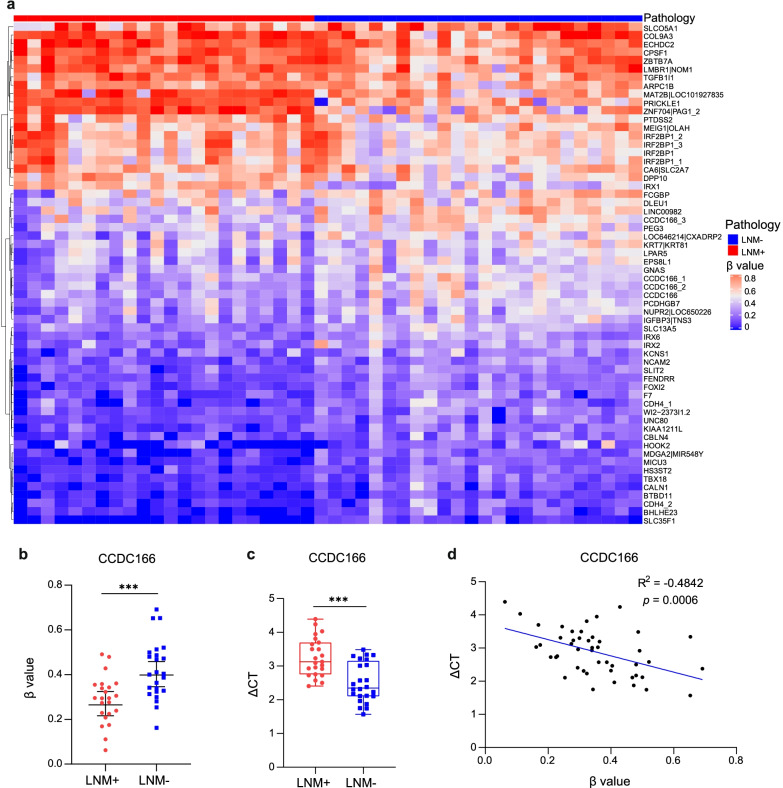
Discovery of DNA methylation markers to detect LNM in EGC tissue. **a** Unsupervised hierarchical clustering of 60 methylation markers differentially methylated between positive lymph node metastatic samples (LNM+, *n* = 23) and negative lymph node metastatic samples (LNM−, *n* = 24) in the discovery cohort. The *β*-value represented the methylation level of markers. A *β*-value of zero represents no methylation, whereas 1 represents full methylation. **b** Methylation level distributions of CCDC166 between positive lymph node metastatic samples (LNM+, *n* = 23) and negative lymph node metastatic samples (LNM−, *n* = 24) as represented by *β*-value from genome-wide methylation sequencing in the discovery cohort. **c** Methylation level distributions of CCDC166 between positive lymph node metastatic samples (LNM+, *n* = 23) and negative lymph node metastatic samples (LNM−, *n* = 24) as represented by Δ Ct values from qPCR-based methylation analysis in the discovery cohort. The data are shown as median with 95% confident intervals. Statistical significance was assessed using a non-paired *t* test (two-tailed). *p < 0.05, **p < 0.01, and ***p < 0.001. **d** Methylation level of CCDC166 from genome-wide methylation sequencing was reversely correlated to Δ Ct values from qPCR-based methylation assay in 10 paired discovery samples. Pearson’s test was used

A schematic workflow of the study design is shown in Fig. 1. To identify DNA methylation markers that are LNM-specific in EGC, we first performed a genome-wide methylation analysis (covering more than 3.34 million CpG sites) on 23 lymph node metastasis positive (LNM+) and 24 lymph node metastasis negative (LNM−) FF tissue samples. A total of 1366 differential methylation CpG sites were found (Additional file 1: Figure S2, FDR < 0.05 and *β*-value difference ≥ 0.2). Based on the methylation sites, we further identified 60 differential methylated regions (hereafter referred to as the “markers”) by using co-methylation region analysis as previously reported [20]. An unsupervised heretical clustering showed a clear differential pattern between the LNM+ and LNM− patients (Fig. 2a). Of the 60 candidate methylation markers, 40 markers were hypomethylated in the LNM+ group including markers of *LAPR5*, *DLEU1*, *FCGBP*, *CBLN4*, *GNAS*, *PCDHGB7*, *NUPR2|LOC650226*, *LOC646214|CXADRP2*, *EPS8L1*, *KCNS1*, *CCDC166*, *IRX6*, *FENDRR*, *SLC13A5*, *HOOK2*, *PEG3*, *UNC80*, *KIAA1211L*, *FOXI2*, *NCAM2*, *SLIT2*, *WI2-237311.2*, *IGFBP3|TNS3*, *BTBD11*, *MICU3*, *F7*, *MDGA2|MIR548Y*, *HS3ST2*, *LNC00982*, *BHLHE23*, *IRX2*, *SLC35F1*, *TBX18*, *CALN1*, *KRT7|KRT81*, two *CDH4* gene regions, and four *CCDC166* gene regions. There were 20 hypermethylated markers including *MEIG1|OLAH*, *PDTSS2*, *TGFB1L1*, *ZBTB7A*, *IRX1*, *SLCO5A1*, *CA6|SLC2A7*, *ECHDC2*, *COL9A3*, *ARPC1B*, *LMBR1|NOM1*, *CPSF1*, *DPP10*, *ZNF704|PAG1*, *MAT2B|LOC101927835*, *PRICKLE1*, and four *IRF2BP1* gene regions (Fig. 2a).

Our primary goal was to develop a simple methylation-specific qPCR assay for LNM status determination [21]. The 60 markers were further validated technically using the same FF samples by a qPCR approach. Among these markers, 50 markers showed consistent methylation patterns between sequencing and methylation-specific qPCR analysis, and significantly distinguished LNM+ from LNM− in the same samples. However, 10 markers were excluded due to failed technical validation with inconsistent methylation pattern between the two assays (Fig. 2b–d, Additional file 1: Figures S3 and S4). These results suggested that these markers and qPCR-based assays were reliable and could be used for large-scale cohort analysis.

### Development and validation of a 3-marker methylation model for LNM diagnosis

**Fig. 3:**
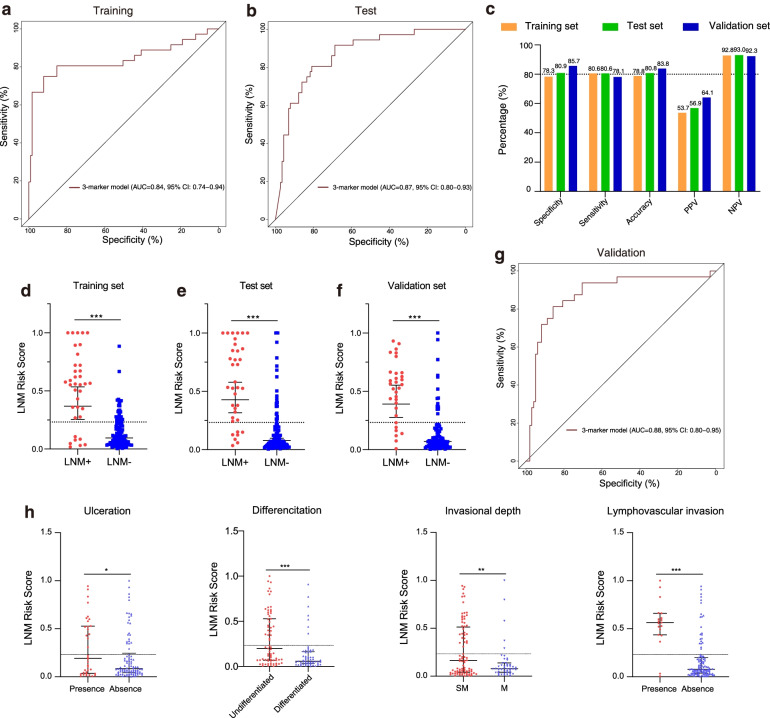
Methylation diagnostic model development and validation for lymph node detection in EGC. **a, b** ROC curves of the 3-marker methylation model in the training and test set, respectively. **c** The sensitivity, specificity, and accuracy of the model were determined by the cutoff value (0.2327) in the training and test set in the model development cohort and independent validation cohort, respectively. **d, e** LNM risk score of the 3-marker methylation model between LNM+ EGC samples (*n* = 36) and LNM− EGC samples (*n* = 115) in the training and test set. The dotted line shows the cutoff value (0.2327) to distinguish LNM+ from LNM− samples. **f** LNM risk score of the 3-marker methylation model between LNM+ samples (*n* = 32) and LNM− EGC samples (*n* = 98) in the validation cohort. **g** ROC curves of the 3-marker methylation model in the validation cohort. **h** LNM risk score of the 3-marker methylation model in different clinicopathological features including the ulcerative type, differentiation, invasional depth, and lymphovascular invasion status. The data are shown as median the interquartile range. Statistical significance was assessed using an unpaired *t* test (two-tailed). *p < 0.05, **p < 0.01, ***p < 0.001

Since in a practical clinical setting, the EGC sample acquired is endoscopic sectioned FFPE samples, we further characterized the 50 methylation markers identified from FF samples by the same qPCR assays in a model development cohort which consisted of 302 FFPE EGCs. To improve the assay diagnostic efficiency and reduce marker redundancy, the least absolute shrinkage and selection operator (LASSO) algorithm was used to determine the minimum number of markers required for maintaining stable diagnostic power and select the corresponding top markers from the 50 candidates. A marker number of five was used for further analysis, and the resulted top 5 markers were subjected for further model development. Methylation models containing any 1–5 markers were iteratively constructed using logistic regression algorithm. By comparing the performance and the performance consistency in 100 random splits of datasets with a train—test ratio of 1:1, a 3-marker methylation model was derived. The 3-marker methylation model, comprising of *GNAS*, *FCGBP*, and *CCDC166*, achieved high AUCs of 0.84 (95% CI 0.74–0.94) and 0.87 (95% CI 0.80–0.93) in the training and test sets, respectively (Fig. 3a, b, Additional file 1: Figure S5a and S5b). The model showed consistent specificities of 78.3% and 80.9%, sensitivities of 80.6% and 80.6%, and accuracies of 78.8% and 80.8% in the training and test datasets, respectively (Fig. 3c). Notably, LNM+ patients showed significantly higher LNM risk scores, calculated from the model, than LNM− patients in both training and test sets (Fig. 3d, e, *p* < 0.001).

The model was further validated in an independent cohort consisting of 30 LNM+ and 98 LNM− patients. It achieved an AUC of 0.88 (95% CI 0.80–0.95), sensitivity of 78.1%, specificity of 85.7%, and accuracy of 83.8% (Fig. 3c, g, Table 2, Additional file 1: Figure S5c). Consistent with the results from the model development cohort, the model showed a significantly higher LNM risk score in the LNM+ patients as compared to LNM− patients (Fig. 3f). We then assessed whether risk scores were associated with clinical characteristics. We found that the LNM risk scores were significantly higher in patients with ulceration, undifferentiation, submucosal invasion, and lymphovascular invasion in the validation cohort (Fig. 3h), indicating the LNM risk scores were associated with the known reported LNM risk factors. On the other hand, the risk score did not vary significantly in EGC patient groups of different age, gender, tumor size, and tumor location (Additional file 1: Figure S6). Taken together, the 3-gene methylation model showed an accurate and robust performance in discrimination for LNM in EGC.

**Table 2:** Characteristics of the 3 methylation markers and their coefficients in EGC LNM diagnosis

Chromosome location	Reference gene	Coefficients	SE	*Z* value	*p* value
Intersect		− 9.462	0.710	− 5.533	0
chr20: 57429888–57429996	GNAS	1.311	0.330	3.978	3.14E−08
chr19: 40421516–40421618	FCGBP	− 0.047	0.039	− 1.195	6.95E−05
chr8: 144790098–144790219	CCDC166	0.652	0.275	2.374	0.0176

SE, standard error of coefficients; *Z*, value Wald Z-statistic value

### The 3-marker methylation model outperformed CT imaging and clinicopathological features for LNM diagnosis

**Fig. 4:**
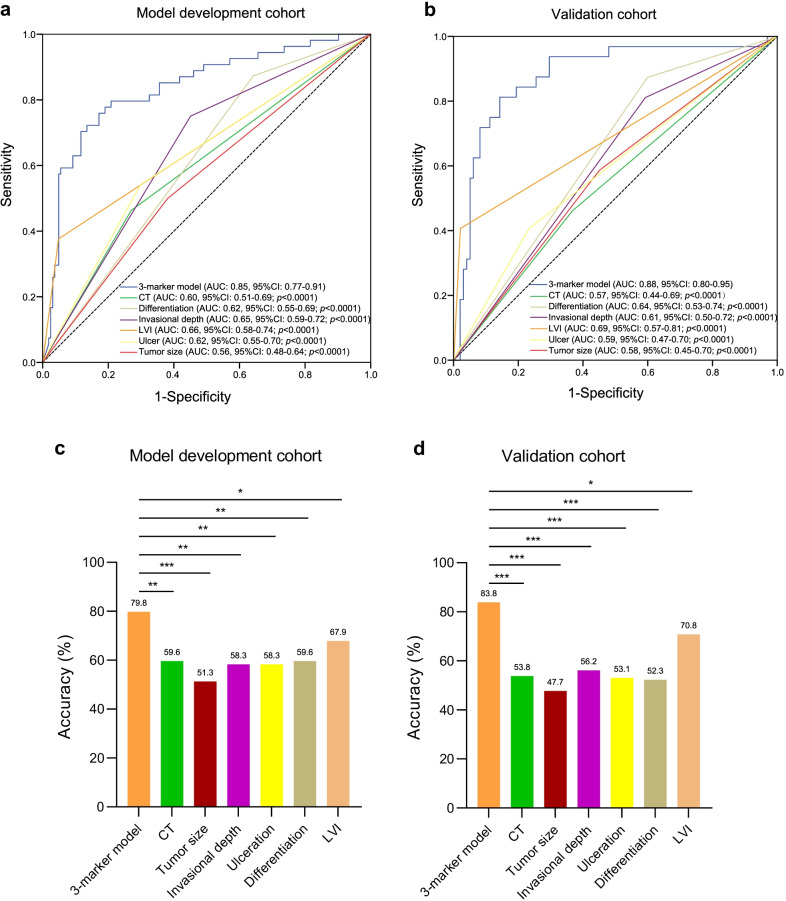
Performance of the 3-marker methylation model compared to preoperative CT imaging and clinicopathological features. **a, b** ROC curves of 3-marker methylation model as compared to those of CT imaging and clinicopathological features including tumor size, lymphovascular invasion, invasional depth, ulceration, and differentiation type in the model development and validation cohorts, respectively. Comparison of AUC values was conducted by DeLong test. **c, d** The accuracy of the 3-marker methylation model as compared to CT imaging and individual clinicopathological features in the model development and validation cohorts, respectively. Statistical significance was assessed by *χ*^2^ test. *p < 0.05, **p < 0.01, ***p < 0.001

In standard clinic settings, CT imaging and clinicopathological factors are used routinely to diagnose LNM and to assess the clinical N stage in patients with EGC before radical treatment. It is well known that clinicopathological features including tumor size, lymphovascular invasion, invasional depth, ulceration, and differentiation type are well-established predictor for the incidence of nodal metastasis for EGC [5, 23]. A univariate analysis was performed for each variable in the model development cohort. Variables of age of under 60 years old (OR 2.559, 95% CI 1.447–4.525, *p* = 0.001), submucosal invasion (OR 3.365, 95% CI 2.008–6.578, *p* < 0.001), tumor size larger than 20 mm (OR 1.625, 95% CI 1.044–2.538, *p* = 0.032), undifferentiated type (OR 3.878, 95% CI 1.834–8.200, *p* < 0.001), lymphovascular invasion (OR 11.950, 95% CI 5.525–25.825, *p* < 0.001), ulceration (OR 2.758, 95% CI 1.603–4.744, *p* < 0.001) were the risk factors significantly associated with LNM. Compared to these risk factors, the 3-marker methylation model indicated significantly higher OR value (OR 16.131, 95% CI 8.289–31.392, *p* < 0.001) (Table 3). Accordingly, we compared the performance of the 3-marker methylation model with CT imaging and these clinicopathological features for EGC LNM diagnosis. Of interest, we found that the diagnostic performance of the 3-marker methylation model (AUC 0.85, 95% CI 0.77–0.91) was significantly higher than CT imaging (AUC 0.60, 95% CI 0.51–0.69; *p* < 0.0001), differentiation (AUC 0.62, 95% CI 0.55–0.69; *p* < 0.0001), invasional depth (AUC 0.65, 95% CI 0.59–0.72; *p* < 0.0001), lymphovascular invasion (AUC 0.66, 95% CI 0.58–0.74; *p* < 0.0001), ulceration (AUC 0.62, 95% CI 0.55–0.70; *p* < 0.0001), and tumor size (AUC 0.56, 95% CI 0.48–0.64; *p* < 0.0001) in the model development cohort (Fig. 4a). In the independent validation cohort, this model also achieved a better performance (AUC of 0.88, 95% CI 0.80–0.95), as compared to CT imaging (AUC 0.57, 95% CI 0.44–0.69; *p* < 0.0001), differentiation (AUC 0.64, 95% CI 0.53–0.74; *p* < 0.0001), invasional depth (AUC 0.61, 95% CI 0.50–0.72; *p* < 0.0001), lymphovascular invasion (AUC 0.69, 95% CI 0.57–0.81; *p* < 0.0001), ulceration (AUC 0.59, 95% CI 0.45–0.70; *p* < 0.0001), and tumor size (AUC 0.58, 95% CI 0.45–0.70; *p* < 0.0001) (Fig. 4b).

**Table 3:** Univariate and multivariate logistic regression of LNM in the modeling cohort

Characteristics	Univariate analysis		Multivariate analysis	
	OR (95% CI)	*p* value	OR (95% CI)	*p* value
Age, years (≤ 60 vs. > 60)	2.56 (1.45–4.53)	0.001		
Gender (Male vs. Female)	1.27 (0.74–2.17)	0.368		
Tumor size, mm (> 20 vs. ≤ 20)	1.63 (1.04–2.54)	0.032		
Differentiation (Undifferentiated vs. Differentiated)	3.88 (1.83–8.20)	< 0.001	3.85 (1.94–7.67)	< 0.001
Invasional depth (SM vs. M)	3.37 (2.01–6.58)	< 0.001	2.48 (1.41–4.36)	0.002
*Tumor location*				
Low versus Upper	1.37 (0.57–3.27)	0.486		
Middle versus Upper	0.64 (0.32–3.13)	0.676		
LVI (Presence vs. Absence)	11.95 (5.53–25.83)	< 0.001	11.30 (5.40–23.64)	< 0.001
Ulceration (Presence vs. Absence)	2.76 (1.60–4.74)	< 0.001	2.36 (1.39–4.00)	0.001
3-Marker methylation (Risk score)	16.13 (8.29–31.39)	< 0.001		< 0.001

OR, odds ratio; CI, confidence interval; M, mucosa; SM, submucosa; LVI, lymphovascular invasion

Accordingly, we compared the performance of the 3-marker methylation model with CT imaging and these clinicopathological features for EGC LNM diagnosis. Of interest, we found that the diagnostic performance (AUC) of the 3-marker methylation model (0.85 and 0.88) was significantly superior than diagnostic model based on CT imaging (0.60 and 0.57), tumor differentiation (0.62 and 0.64), tumor invasional depth (0.65 and 0.61), tumor lymphovascular invasion (0.66 and 0.69), ulceration (0.62 and 0.59), and tumor size (0.56 and 0.58) in the model development and validation cohort, respectively (Fig. 4a, b). The 3-marker methylation model showed significantly higher accuracies (79.8% and 83.8%) than diagnostic model based on CT imaging (59.6% and 53.8%), tumor differentiation (59.6% and 52.3%), tumor invasional depth (58.3% and 56.2%), tumor lymphovascular invasion (67.9% and 70.8%), ulceration (58.3% and 53.1%), and tumor size (51.3% and 47.7%) in the two cohorts, respectively (Fig. 4c, d). The sensitivity and specificity of the 3-marker methylation model were also significantly higher than diagnostic models based on CT imaging or individual clinicopathological features (Additional file 1: Figure S7), with approximately twofold higher sensitivities as compared to CT-based diagnostics (80.6% vs. 41.7% and 78.1% vs. 40.6% in the model development and validation cohort, respectively) (Additional file 1: Figure S7c and S7d).

### An integrated model combining methylation and clinicopathological features further improved the LNM diagnostic performance

**Fig. 5:**
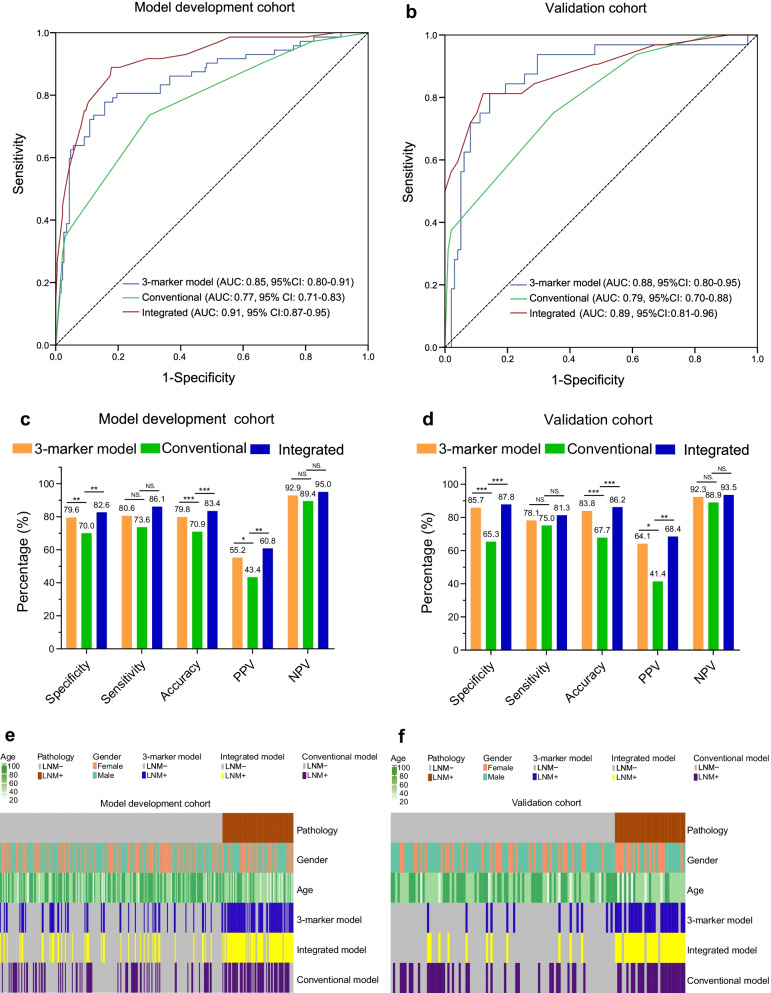
Performance comparison between the conventional model, 3-marker methylation model, and integrated model. **a, b** ROC curve of the 3-marker methylation model and integrated model as compared the conventional model in the model development and validation cohorts, respectively. Comparison of AUC values was conducted by Hanley and McNeil tests. **c, d** The sensitivity, specificity, and accuracy of the 3-marker methylation model and integrated model as compared the conventional model in the model development and validation cohorts, respectively. **e, f** Distribution of predicted LNM status in EGC using 3-marker model, Integrated model and conventional model in the model development and validation cohorts, respectively. Statistical significance was assessed by *χ*^2^ test. *p < 0.05, **p < 0.01, ***p < 0.001, NS., not statistically significant

To evaluate the performance of the 3-marker methylation model and the clinicopathological characteristic-based model (i.e., the conventional model [17, 18]), the risk factors as identified by previous univariate analysis were used in multivariate analysis to select independent LNM predictors (Table 3 and Additional file 2: Table S3) and these predictors, including lymphovascular invasion (OR 11.30, 95% CI 5.40–23.64, *p* < 0.001), submucosal invasion (OR 2.48, 95% CI 1.41–4.36, *p* = 0.002), ulceration (OR 2.36, 95% CI 1.39–4.00, *p* = 0.001), and differentiation (OR 3.85, 95% CI 1.94–7.67, *p* < 0.001), were further used for development of a conventional model. We developed a conventional model based on informative pathological features as reported before [22]. However, the performance of the conventional model was inferior to the 3-marker methylation model, with lower AUCs in the model development cohort (0.77, 95% CI 0.71–0.83 vs. 0.85, 95% CI 0.80–0.91, *p* = 0.0805) and the validation cohort (0.79, 95% CI 0.70–0.88 vs. 0.88, 95% CI 0.80–0.95, *p* = 0.1250), respectively (Fig. 5a, b). Compared to the conventional model, the 3-marker methylation model achieved higher specificity (79.6% vs. 70.0% and 85.7% vs. 65.3%) and accuracy (79.8% vs. 70.9% and 83.8% vs. 67.7%) with comparable sensitivity (80.6% vs. 73.6% and 78.1% vs. 75.0%) in the model development and validation cohorts, respectively (Fig. 5c, d). Diagrams illustrating the predicted results of both the 3-marker methylation model and conventional model as compared to pathology for the same persons in method development and validation cohorts are shown in Fig. 5e, f. For the same patients having LNM, the 3-marker methylation model and conventional model showed a high concordance with the 3-marker methylation model identified additionally more cases. More importantly, the 3-marker methylation model helped more patients without LNM to avoid over treatment.

To explore whether the diagnostic accuracy of the 3-marker methylation model could be enhanced by combining clinicopathological features, we built an integrated model within the model development cohort using independent predictors of LNM, which included 3-marker methylation model (OR 17.616, 95% CI 9.144–33.937, *p* < 0.001), submucosal invasion (OR 2.602, 95% CI 1.345–5.037, *p* = 0.005), differentiation (OR 3.863, 95% CI 1.733–8.609, *p* = 0.001), ulceration (OR 2.692, 95% CI 1.443–5.022, *p* = 0.002), and lymphovascular invasion (OR 9.956, 95% CI 4.144–23.917, *p* < 0.001), as shown in Additional file 2: Table S4. The integrated model showed improved AUCs of 0.91(95% CI 0.87–0.95, *p* < 0.0001) and 0.89(95% CI 0.81–0.96, *p* = 0.0079), specificities of 82.6% and 87.8%, and accuracies of 83.4% and 86.2% and compatible sensitivities of 86.1% and 81.3% as compared to the methylation model and conventional model in the model development and validation cohorts, respectively (Fig. 5c–f).

### Both the 3-marker methylation model and the integrated model have the potential to reduce overtreatment on LNM− EGC patients

**Fig. 6:**
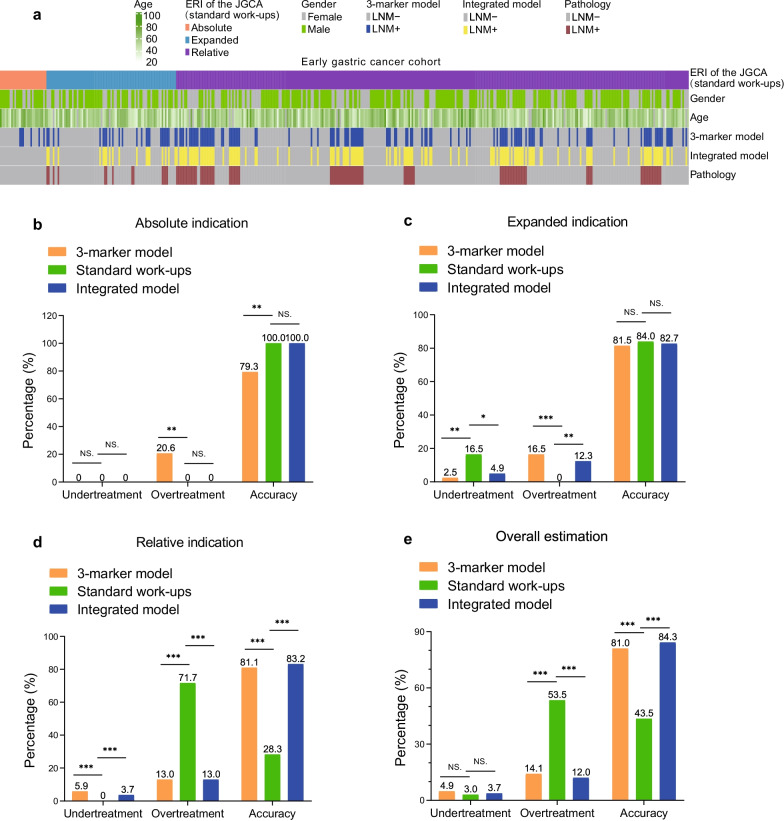
Performance of the 3-marker methylation model and integrated model as compared to the standard workups. **a** Treatmental modalities of EGC patients in the two cohorts as guided by the 3-marker methylation model and the integrated model as compared to standard workups (JGCA). Absolute indication for endoscopic resection (ERI), differentiated EGC confined to the mucosa without ulcerative findings and with a tumor diameter ≤ 2 cm. Expanded indication for endoscopic resection, mucosal gastric cancers of the following categories: (i) differentiated-type, ulceration (absence), diameter > 2 cm; (ii) differentiated-type, ulceration (presence), diameter ≤ 3 cm; and (iii) undifferentiated-type, ulceration (absence), diameter ≤ 2 cm). Relative indication for endoscopic resection, EGC tumors that do not fulfill the absolute or expanded indications. **b**–**d** The undertreatment rate, overtreatment rate, and accuracy of the 3-marker methylation model and the integrated model compared to standard workups in patients as indicated for absolute indication, expanded indication, and the relative indication of endoscopic resection (*n* = 29, 81 and 322). **e** A overall estimation of undertreatment rate, overtreatment rate, and accuracy analysis of the 3-marker model and the integrated model as compared to the standard workups. Statistical significance was assessed using *χ*^2^ test. *p < 0.05, **p < 0.01, ***p < 0.001, NS., not statistically significant

The treatment modalities of EGC depend on the status of LNM in patients. While ESD has been used as the curative procedure of EGC without LNM, surgical resection of tumors with D1/D2 lymphadenectomy is conducted in patients diagnosed with LNM. However, the identification of LNM is not sufficient under current standard workups (The Japan Gastroenterological Endoscopy Society and Japanese Gastric Cancer Association (JGCA) guidelines) [5, 23]. To test whether the 3-marker methylation model can augment LNM diagnosis accuracy and treatment precision, we compared the clinical utilities of the 3-marker methylation model and the integrated model to current standard workups in overall 432 surgically resected specimens. For patients with the absolute indication of ESD in our cohorts (*n* = 29), the 3-marker methylation model and integrated model resulted in 79.3% and 100% diagnostic accuracy, 0.0% undertreatment, and 20.7% and 0.0% overtreatment due to false positive identification, as compared to standard workups of 100.0% accuracy, 0.0% undertreatment and 0.0% overtreatment (Fig. 6a, b). For patients with expanded indication of ESD in our cohort (*n* = 81, 13 of LNM+, and 68 of LNM−), while the overtreatment rate of the 3-marker methylation model and integrated model was slightly higher as compared to standard workups (16.0% and 12.3% vs. 0.0%), the undertreatment rates of our models were significantly lower (2.5% and 4.9% vs. 16.1%) and the overall accuracies were comparable to standard workups (81.5%, 82.7% vs. 84.0%) (Fig. 6a, c). For patients with relative indication (*n* = 322, 91 of LNM+ and 231 of LNM−), the 3-marker methylation model and integrated model showed significantly improved accuracies as compared to standard workups (81.1%, 83.2% vs. 28.3%). Additionally, the 3-marker methylation model and integrated model showed remarkably low overtreatment rates (13.0%, 13.0% vs. 71.74%) (Fig. 6a, d). Since 74.5% of the overall EGC patients are relative indications, the 3-marker methylation model and integrated model have the potential to significantly reduce the overtreatment rate by 39.4% and 41.5% (14.1% and 12.0% vs. 53.5%), respectively, while maintaining a comparable undertreatment rate (4.9% and 3.7% vs. 3.0%) (Fig. 6a, e). Based on our findings, the potential of the methylation model and integrated model integrated in current clinical diagnostic setting was proposed (Additional file 1: Figure S8).

## Discussion

In this study, we performed a comprehensive genome-wide methylation profiling on EGC tissues and identified 60 LNM-specific methylation markers. Derived from these markers, a qPCR-based 3-marker methylation model was developed and validated with large-scale retrospective cohorts, consisting of 302 and 130 tissue samples, respectively. This model was superior to the most commonly used clinicopathological-based conventional tools in diagnosing LNM, as shown in our head-to-head comparison (AUC 0.85 vs. 0.77 in model development cohort and AUC 0.88 vs. 0.79 in validation cohort), while the conventional model we developed using the clinicopathological information showed similar diagnostic power as compared to previous studies (0.84 in the model development cohort and 0.82 in the validation cohort) [16]. The 3-marker methylation model also showed advantageous diagnostic potential as compared to the reported gene expression-based methods, in which a 15-gene signature was used to identify LNM in early stage (T1–T2) gastric cancer with an AUC of 0.76 in training and AUC of 0.74 in the validation set [24]. The results indicate the robustness of DNA methylation as diagnostic biomarker as compared to RNA expression, as DNAs were relatively stable clinical material and DNA methylation profiles may represent a relatively stable long-term programming of the genome and underlying cellular functions, whereas transcription assays only provide a snapshot of the gene expression activity at a specific time point and represent a transient signaling process [13].

To date, few studies have used genome-wide methylation strategy to screen methylation markers for LNM diagnosis in EGC. Wu et al. reported a 14 LNM-related genes classifier derived from 450 K methylation data of gastric cancer in The Cancer Genome Atlas (TCGA) and developed 14 LNM-related genes classifier which showed a median AUC of 0.78 [25]. Our study applied a more comprehensive approach to dissect the methylome associated with LNM in EGC, with more than 3.34 million CpG sites analyzed which accounted for 97.3% of CpG islands in the genome. The de novo marker discovery effort identified some LNM-specific markers that were first reported in EGC, including the 3 markers (*GNAS*, *FCGBP*, and *CCDC166*) used in the methylation model.

Previous studies have shown that DNA methylation levels of imprinted domains of *GNAS* in primary breast cancer, lung cancer, and ovarian cancer are very different from those in normal tissues. It has been shown that *GNAS* promotes breast cancer cell proliferation and epithelial–mesenchymal transformation (EMT) through the *PI3K/Akt/Snail1/E-cadherin* signaling pathway, which may be responsible for the malignant progression and metastasis [26, 27]. The discovery of methylated region was found in the first exon region of *GNAS* which is hypomethylated in LNM + EGC in our study, suggesting that imprinted domains in *GNAS* could play a role in gastric cancer metastatic development as well.

*FCGBP* (Fc fragment of IgG binding protein) has been identified as a metastasis-related gene in colorectal cancer; its down-regulation is an independent risk factor for overall survival and disease-free survival in patients with metastatic colorectal cancer and is significantly associated with the prognosis of those patients [28, 29]. We found that the methylated region of *FCGBP* gene is located in the fifth exon region inside the gene, which may be involved in the regulation of gene expression and affect its function on LNM in gastric cancer. *CCDC166* was found to be highly mutated in signet ring cell carcinoma [30]. The mutant region did not occur within the methylated region we found. It was discovered that the methylated region is located in the first exon region of *CCDC166* and is hypomethylated in LNM+ EGC in our study. Further studies are needed to explore the biological functions and potential regulatory network of these methylation markers in promoting LNM in EGC.

In current clinical settings, endoscopic ultrasound, CT imaging, and clinicopathological features are standard workups for determining the N staging of gastric cancer. As different N staging may lead to different operative management, it is crucial to accurately access the N staging preoperatively. However, preoperative LNM identification is limited with current technologies. Endoscopic ultrasonography was reported with an accuracy of 43%, while CT imaging has an accuracy of 56% [31, 32]. Clinicopathological features can be examined pathologically with endoscopically resected tissues (EMR or ESD) from EGC patients. Patients found with at least one positive pathological feature, such as undifferentiated type, submucosal invasion, lymphatic vascular invasion, or ulceration, are usually recommended for radical surgical procedures [33].

While the incidence of LNM in EGC is about 8%-25%, approximately 69.1% of the patients with EGC undergo radical gastrectomy with a lymphadenectomy according to standard workups [34], indicating the current pathological assessment-based LNM diagnosis procedures are suboptimal that resulted in high rate of overtreatment and unnecessary gastrectomies. CT-positive findings that are largely based on nodule size and/or volume are often accompanied by high false-negative rates [12].

Our 3-marker methylation model demonstrated improved performance over these current conventional methods. We found the LNM risk score calculated from our model was significantly associated with the LNM status in patients but not their age, gender, tumor size, and tumor location. The 3-marker methylation model and integrated model showed significantly improved specificity and low false positive rates, resulting in a remarkable reduction of overtreatment by 39.4% and 41.5% as compared to standard workups; this result suggested a great potential of the assay to reduce unnecessary gastrectomies. However, it is worth pointing out that our study was based on samples that were surgically resected; thus, a large-scale multi-center study with preoperative endoscopic biopsies or endoscopically resected specimens is needed to confirm the robustness and performance of the assay.

## Conclusions

In summary, we have established and validated a novel 3-marker methylation model in a large retrospective cohort, with the intention to improve LNM diagnosis accuracy in EGC. With further developments, we are hopeful that we would integrate it into existing preoperative LNM diagnosis procedures and assist in guiding treatment decision making in EGC patients.

## Supplementary Information

**Additional file 1:** Supplemental Figures.

**Additional file 2:** Supplemental Methods and Tables.

## Data Availability

The datasets used and/or analyzed during the current study are available from the corresponding author on reasonable request.

## Abbreviations

AUC: Area under curve
EGC: Early gastric cancer
LNM: Lymph node metastasis
LNM+: Positive lymph node metastasis
LNM−: Lymph node metastasis negative
EMR: Endoscopic mucosal resection
ESD: Endoscopic submucosal dissection
CT: Computed tomography
PET-CT: Positron emission tomography with CT
FF: Fresh frozen
FFPE: Formalin-fixed paraffin-embedded
M: Mucosal invasion
SM: Submucosal invasion
LVI: Lymphovascular invasion
ERI: Indication of endoscopy resection. Ct: cycle threshold
LASSO: The least absolute shrinkage and selection operator
ROC: Receiver operating characteristic
OR: Odds ratio
