# Supplementary material for: A novel DNA methylation signature associated with lymph node metastasis status in early gastric cancer
Source: Clin Epigenetics. 2022 Feb 3;14:18. doi: 10.1186/s13148-021-01219-x (PMC8811982; doi:10.1186/s13148-021-01219-x)
Supplement: Supplementary file 1 — Additional file 1: Supplemental Figures. [file 13148_2021_1219_MOESM1_ESM.docx]

**Additional file 1**


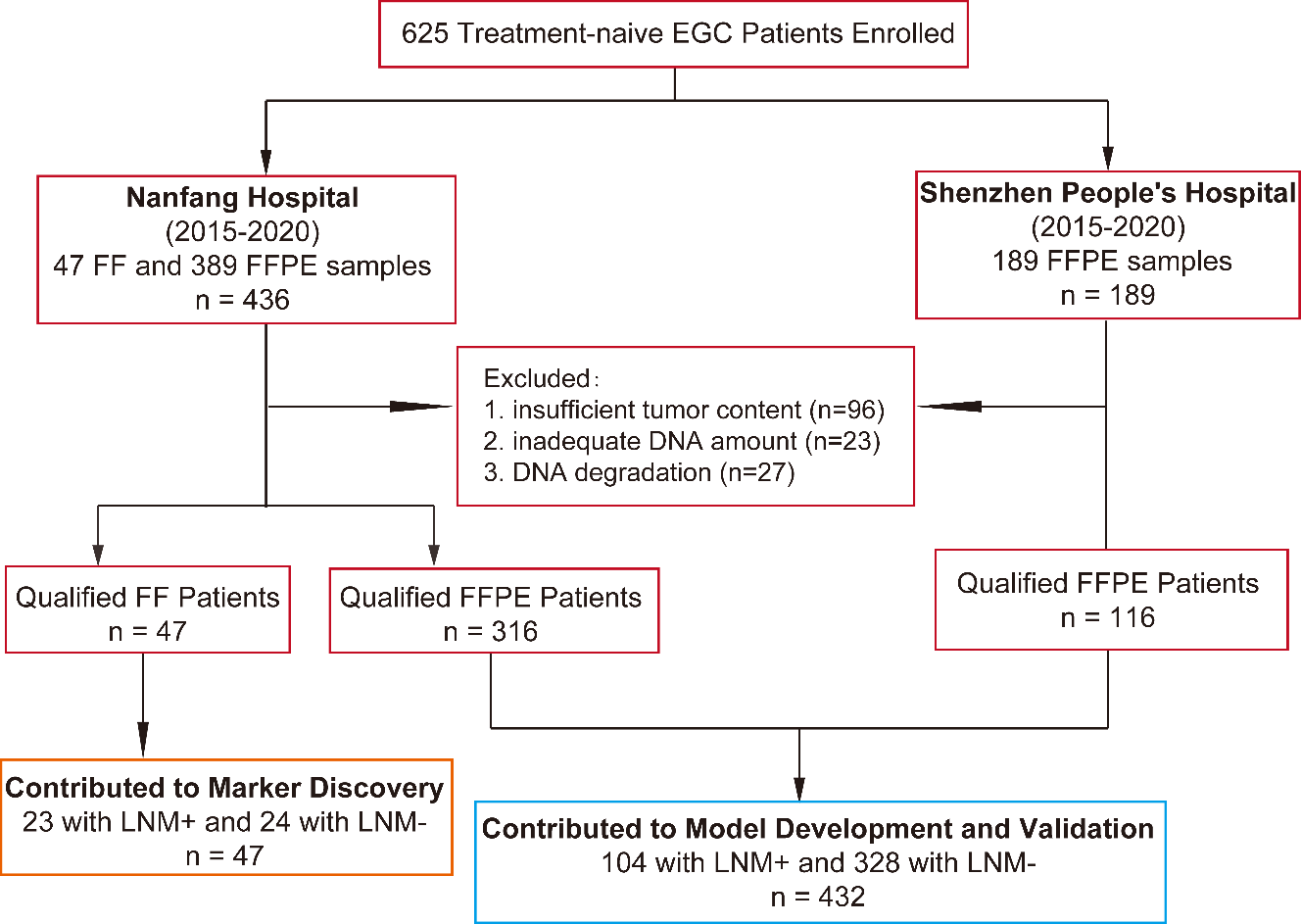


**Figure S1**. Overview of patient recruitment workflow. A total of 625 treatment-naïve EGC patients were enrolled from Nanfang Hospital and Shenzhen People's Hospital between January 2015 and November 2020. 146 samples were excluded from analysis due to failed experimental QC, e.g. insufficient tumor content (n=96), inadequate DNA amount extracted from tumors (n=23), or DNA degradation (n=27). The remaining 479 patient samples were used for marker discovery (47 FF samples), model development and validation (432 FFPE samples).


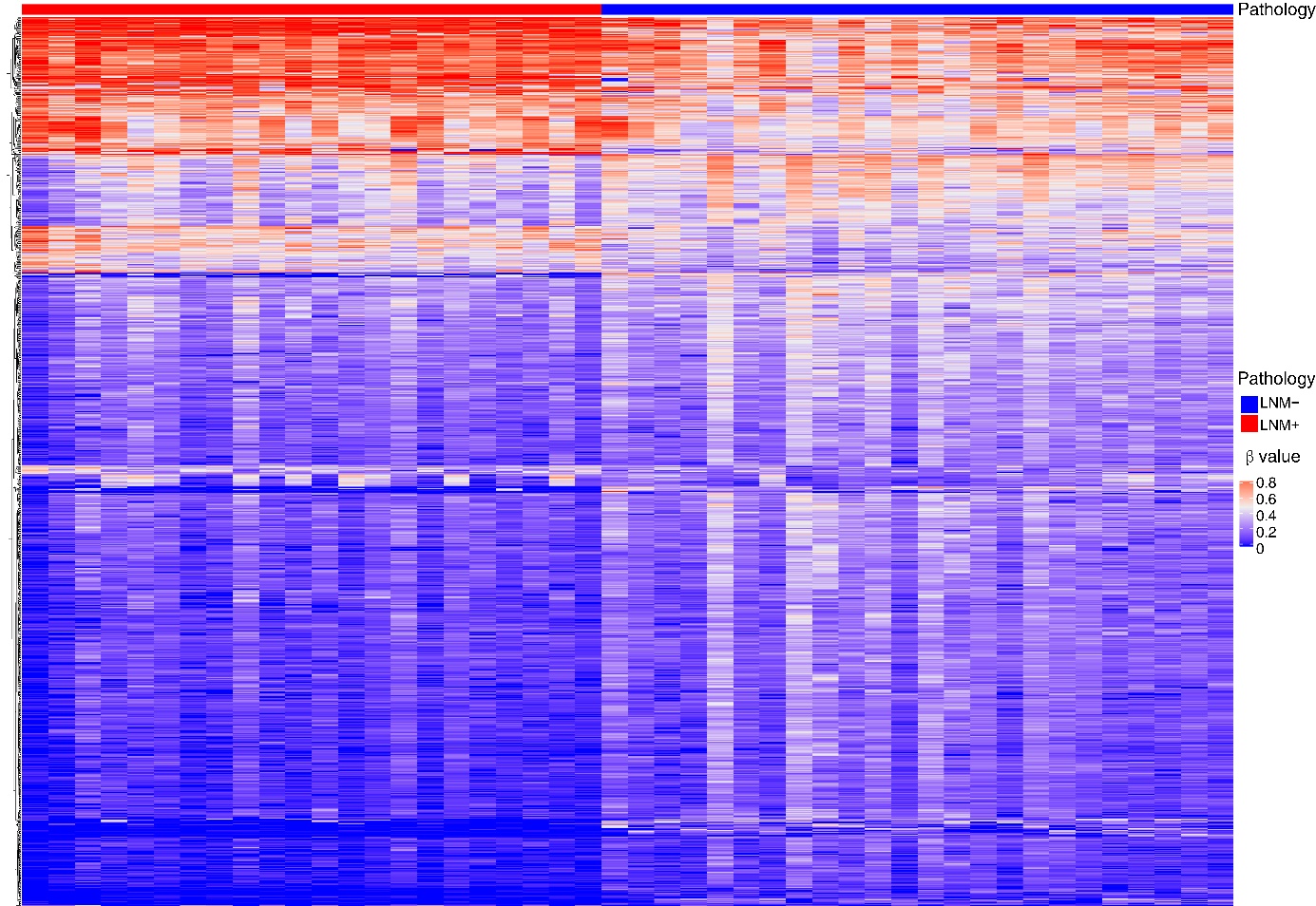


**Figure S2.** Discovery of DNA methylation markers to differentiate LNM+ from LNM- samples. Unsupervised hierarchical clustering of 1366 differentially methylated markers among LNM+ (*n* = 23) and LNM- (*n* = 24) samples in the discovery cohort.


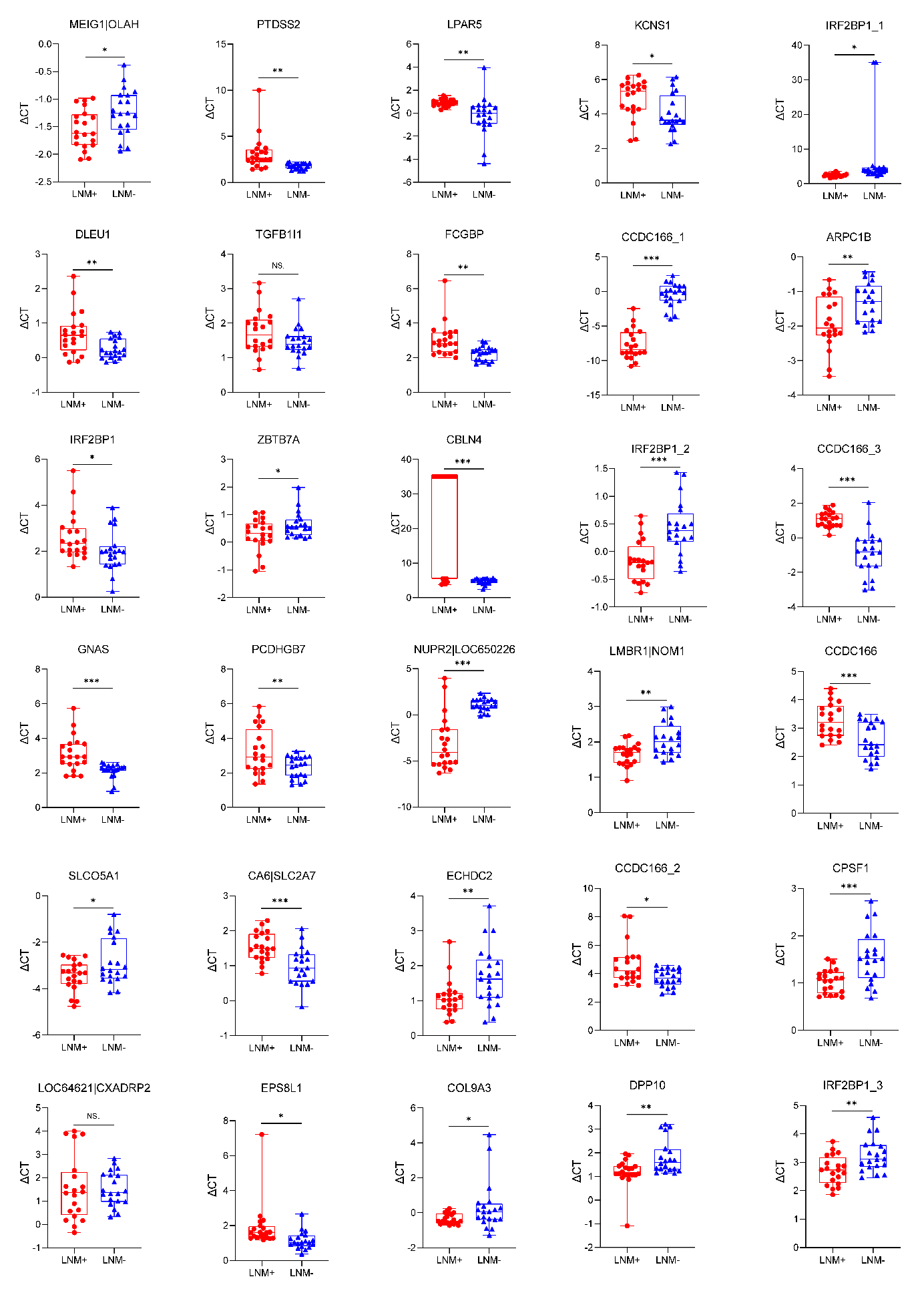


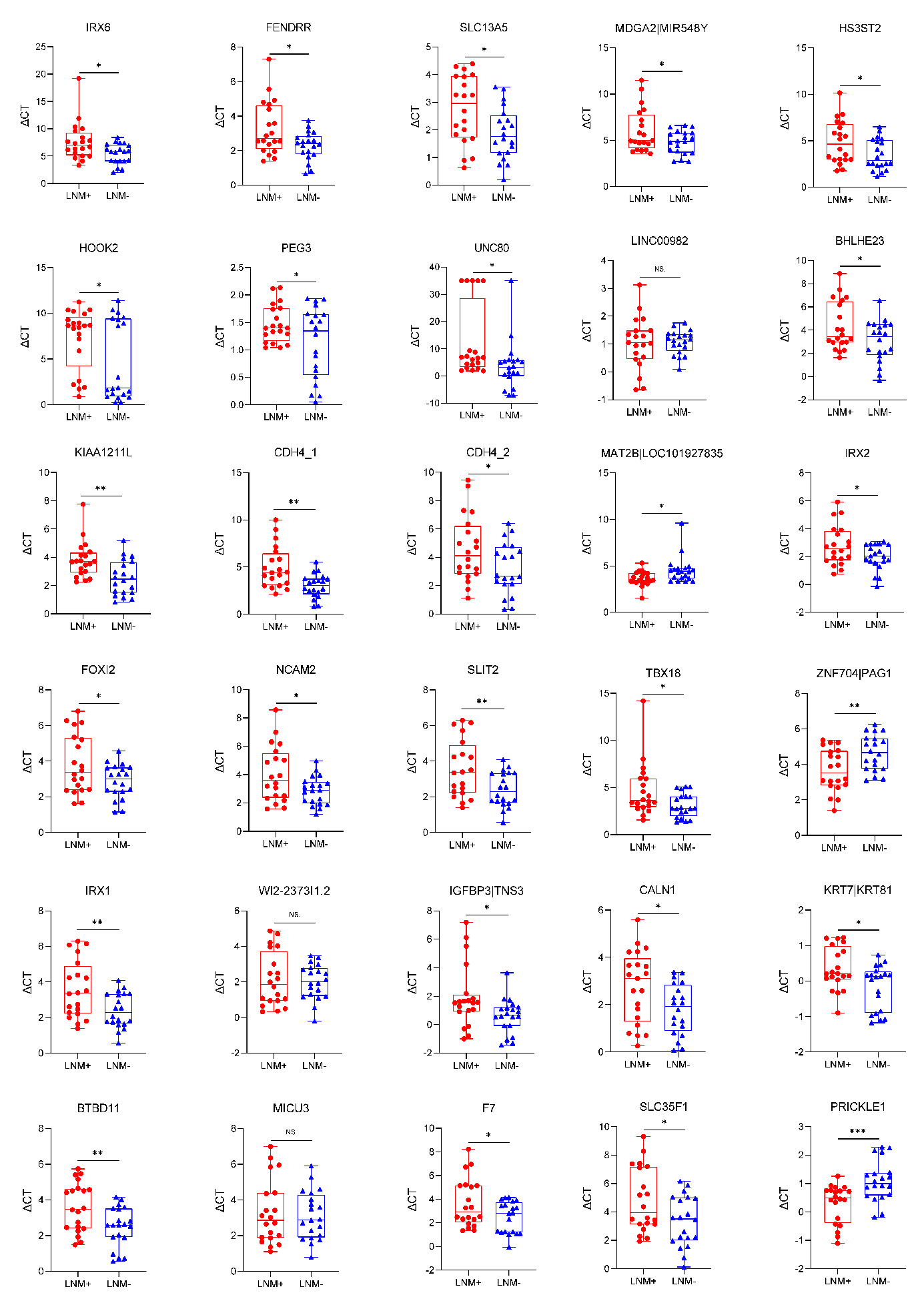


**Figure S3.** Boxplot presenting ΔCt value distribution of 60 targeted methylation regions for technique validation based on methylation specific qPCR among LNM+ (n = 23) and LNM- (n = 24) in the discovery cohort. Methylation levels of the markers were inversely represented by the ΔCt values. Different regions of the same gene are annotated as gene _numbers. An intergenic region is annotated as gene1/gene2, where gene 1 and gene 2 are the two adjacent genes of the intergenic region. The data were presented as median with the interquartile range. Statistical significance was assessed using student t tests (2-tailed). **p* < 0.05, ***p* < 0.01 and ****p* < 0.001.


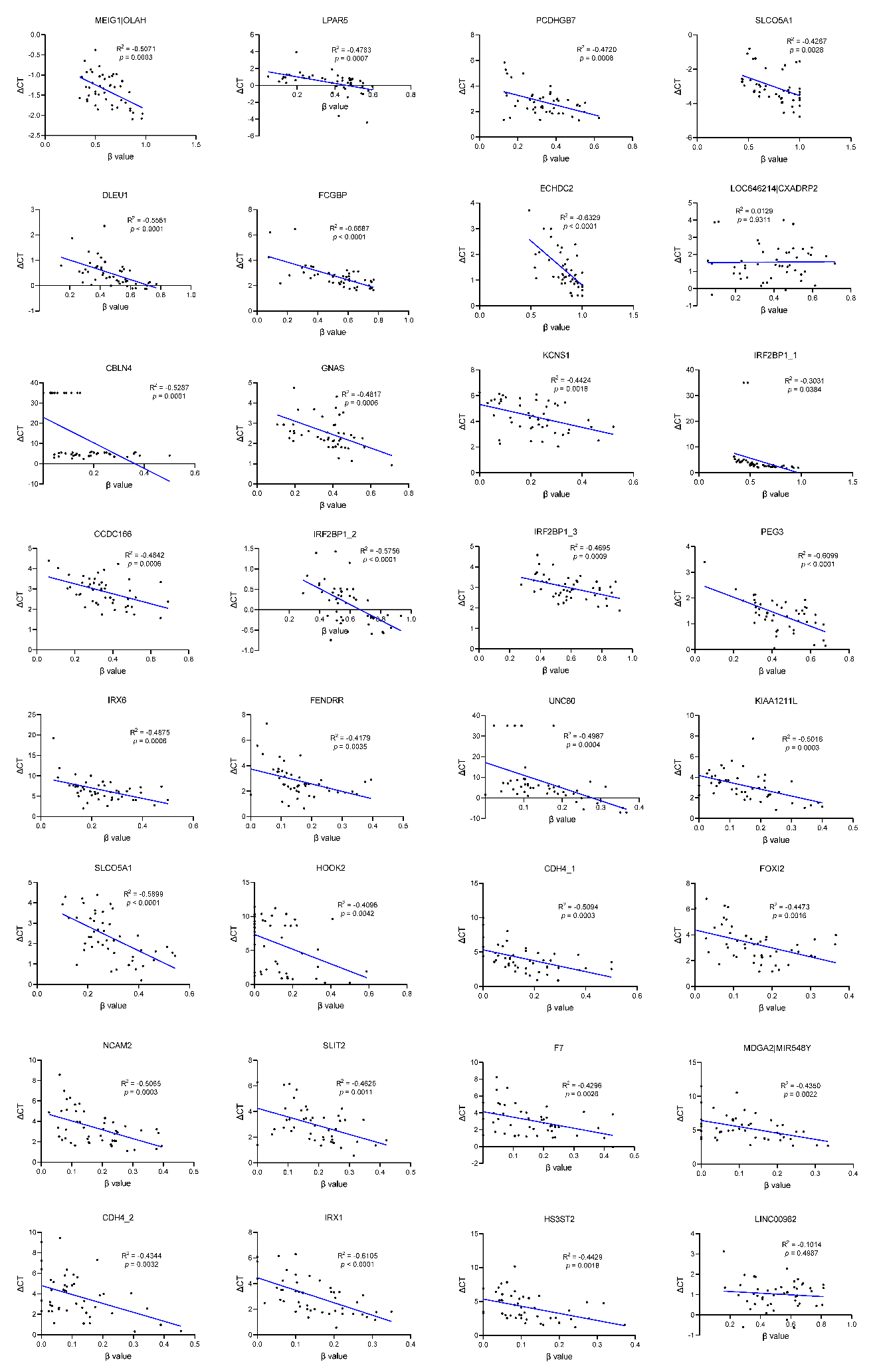


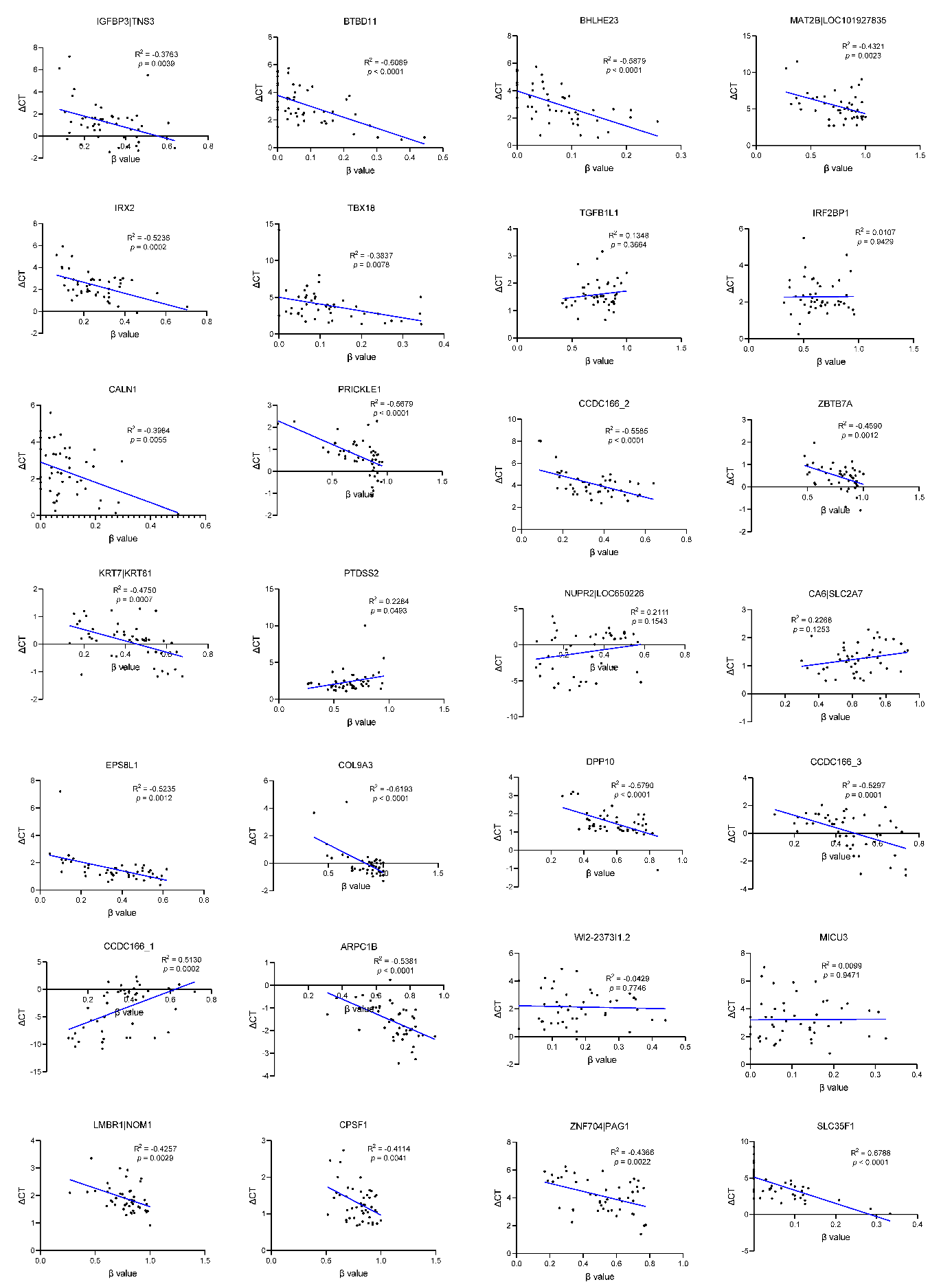


**Figure S4.** The Pearson correlation analysis of the methylation levels of 60 methylation regions between genome-wide sequencing data and qPCR data in 20 EGC patients of the discovery cohort. The detail R^2^ and *p* value were shown in the figure. Pearson’s test was used to analyse statistical significance. Different regions of the same gene are annotated as gene _numbers. An intergenic region is annotated as gene1/gene2, where gene 1 and gene 2 are the two adjacent genes of the intergenic region.


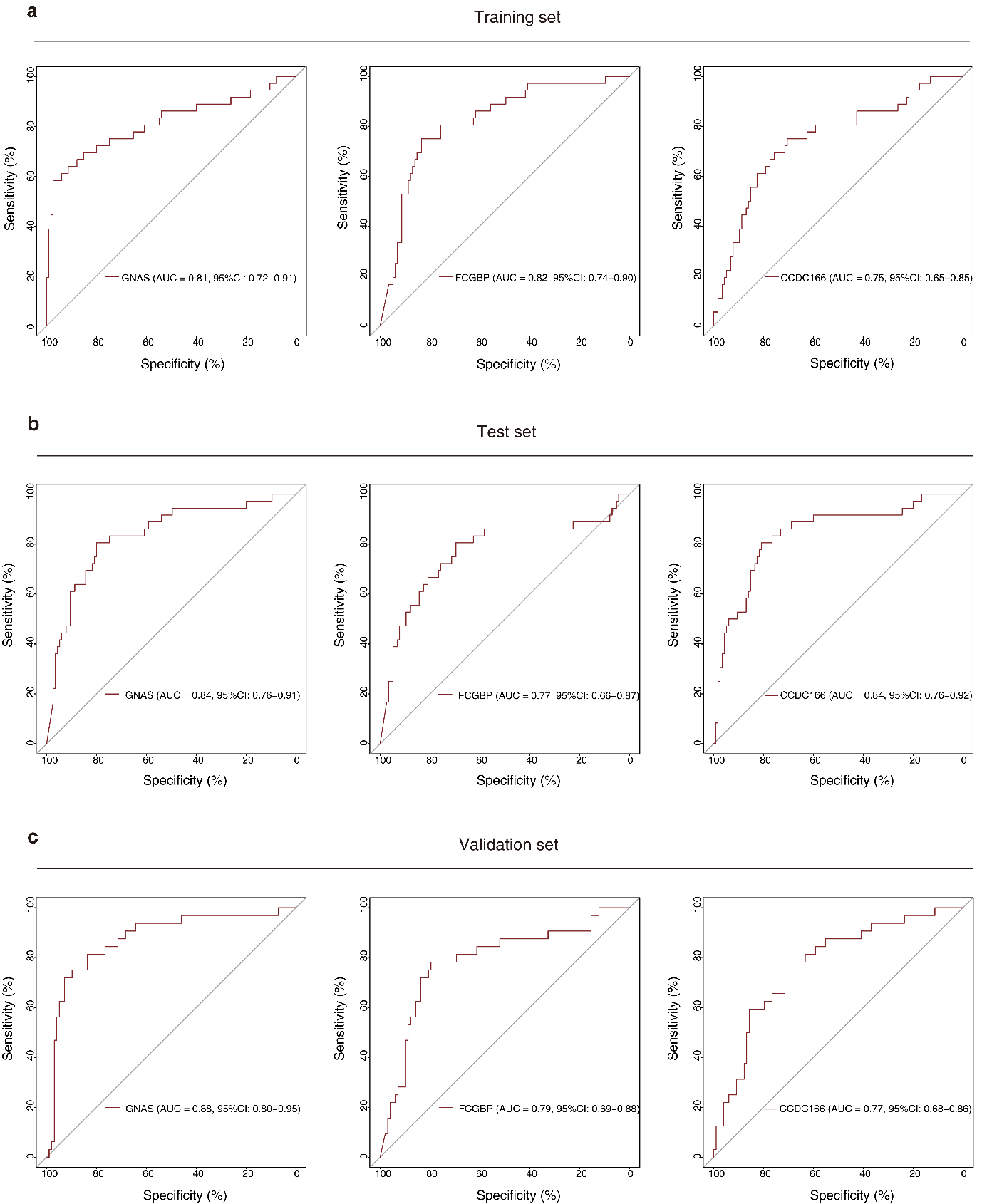


**Figure S5.** The diagnostic efficiency of each marker in the 3-marker methylation model. (**a-c**) ROC curves for each marker in the training, test, and validation sets.


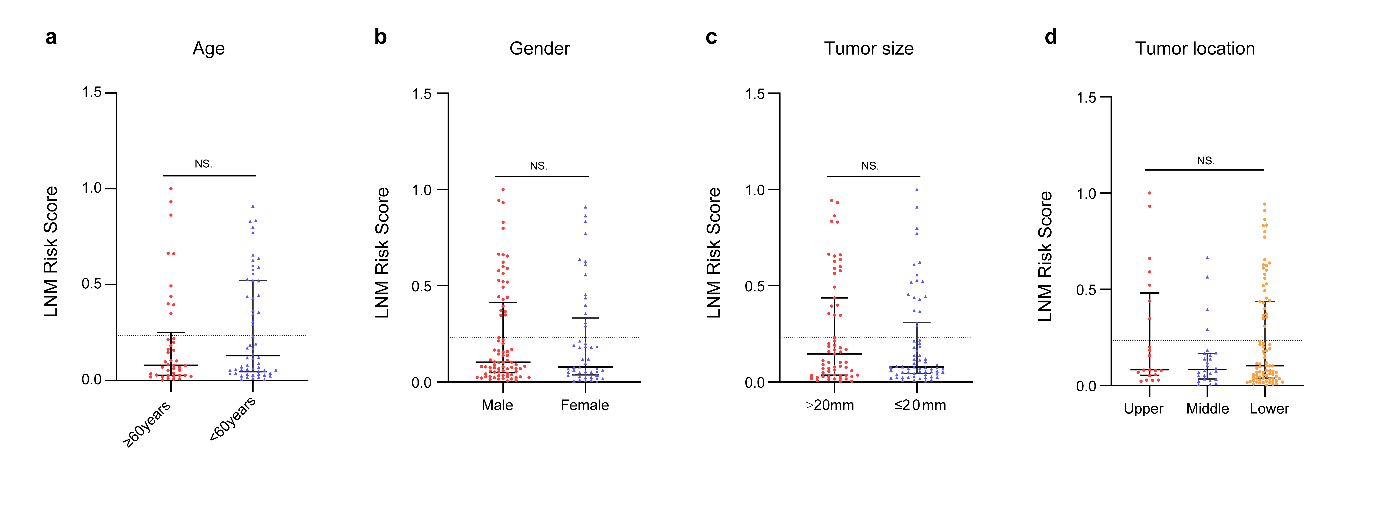


**Figure S6.** The distribution of LNM risk score in different clinical parameters in the validation cohort. **a, b** The distribution of LNM risk score in age (<60 years or ≥60 years) and gender of EGC patients. **c,** **d** The distribution of LNM risk score in tumor size (≤20mm or >20mm) and location (upper, middle and lower) of EGC patients. The dotted line shows the cutoff value (0.2327) to distinguish LNM+ from LNM- samples. The data were presented as median with the interquartile range. Statistical significance was assessed using student t tests (a-c) and 1-way ANOVA followed by Dunnett’s tests (d). NS represents no significance.


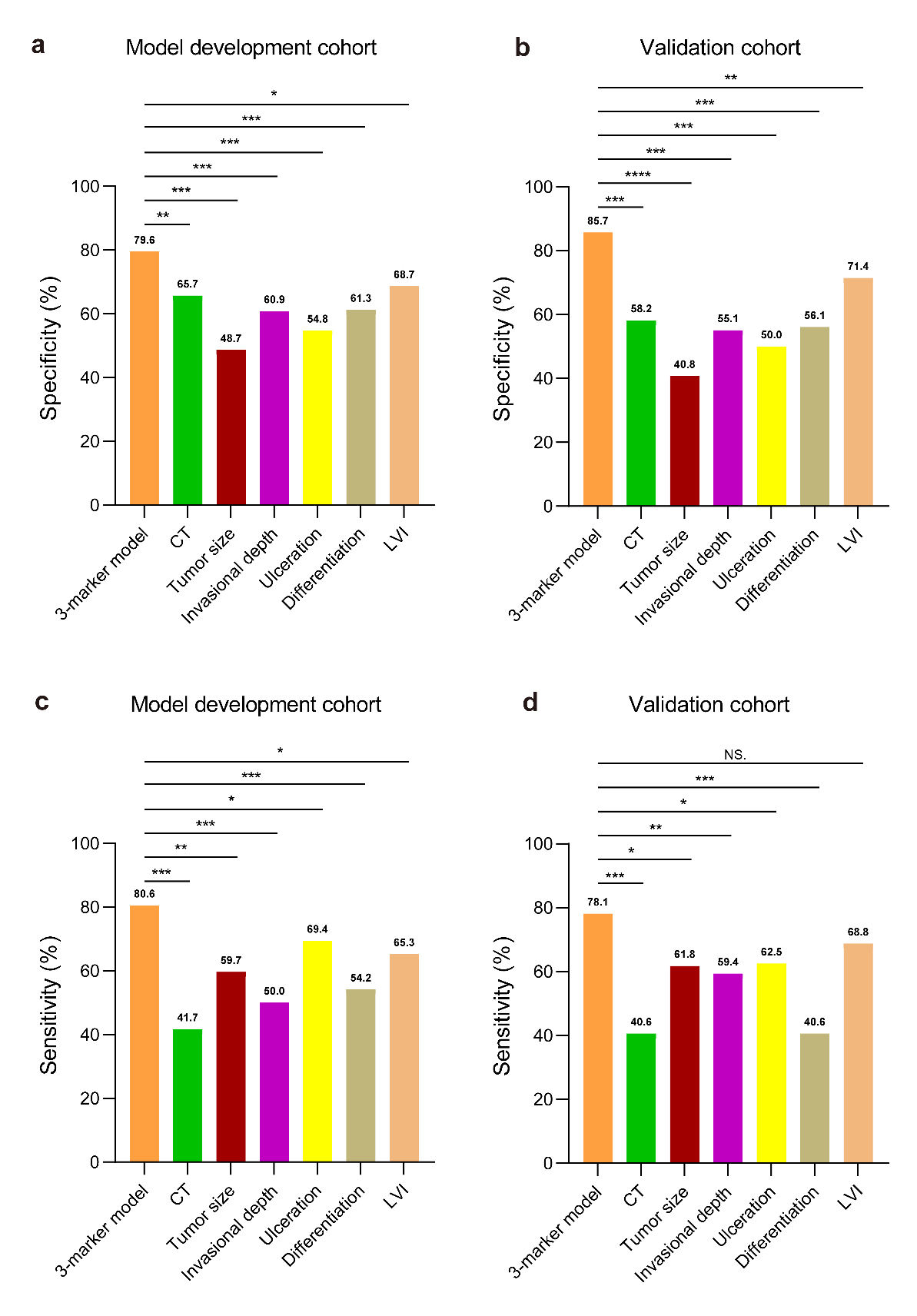


**Figure S7.** The sensitivity and specificity of 3-marker methylation model, preoperative CT imaging and clinicopathological features including tumor size, lymphovascular invasion, invasional depth, ulceration, and differentiation type. **a, b** The sensitivity of 3-marker methylation model in the model development and validation cohorts respectively, in comparison with CT imaging and individual clinicopathological features. **c,** **d** The specificity of 3-marker methylation model in the model development and validation cohorts respectively, in comparison with CT imaging and individual clinicopathological features. Statistical significance was assessed by χ2 test. *LVI* lymphovascular invasion. **p* < 0.05, ***p* < 0.01 and ****p* < 0.001.


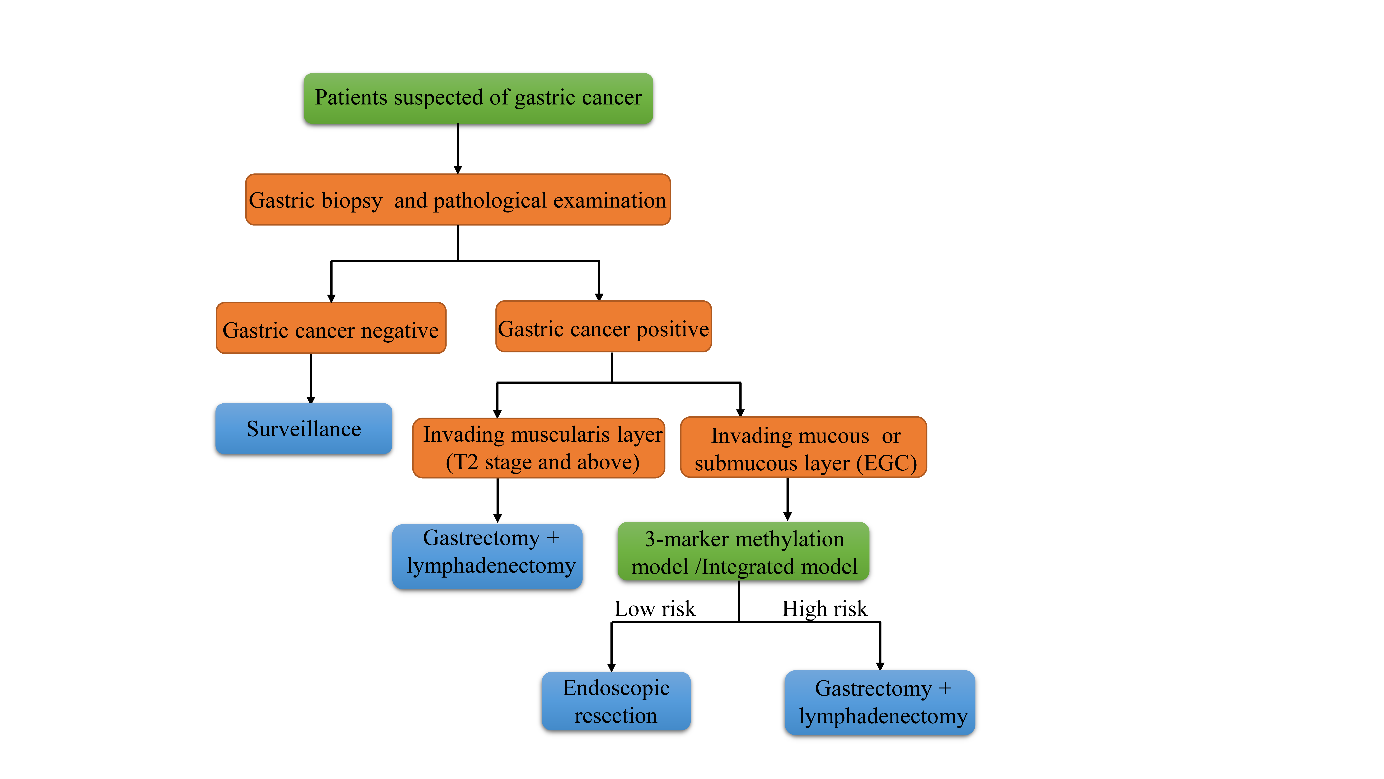


**Figure S8.** Overview of proposed application workflow of the methylation assay integrated in current clinical diagnostic setting. Firstly, qualitative diagnosis was made on the biopsy specimen by pathological examination to determine whether the lesion was cancer as standard workups. For patients diagnosed with tumors infiltrated the muscular is layer (T2 or later stage), gastrectomy and lymphadenectomy were performed following current clinical guidelines. The methylation assay was only applied to those as diagnosed with EGC. Based on the LNM risk score of the methylation model or integrated model, patients with LNM risk score exceeding to cutoff value were recommended for gastrectomy and lymphadenectomy, while patients with the LNM risk score lower than the cutoff value were recommended for endoscopic treatment.
