# Supplementary material for: A novel DNA methylation signature associated with lymph node metastasis status in early gastric cancer
Source: Clin Epigenetics. 2022 Feb 3;14:18. doi: 10.1186/s13148-021-01219-x (PMC8811982; doi:10.1186/s13148-021-01219-x)
Supplement: Supplementary file 2 — Additional file 2: Supplemental Methods and Tables. [file 13148_2021_1219_MOESM2_ESM.docx]

**Additional file 2**

**Methods**

**Genome-wide methylation sequencing and sequencing data analysis**

Briefly, the genomic DNA extracted from tissue was fragmented to 200 bp using the M220 Focused-ultrasonicator™(Covaris, Inc., United States, Catalog No. 500295) following the manufacturer’s protocol. 800 ng of fragmented DNA were input genome-wide methylation library using TruSeq® Methyl Capture EPIC Library Prep Kit (Illumina, United States, Catalog No. FC-151-1002) following the manufacturer’s protocol. The concentration of final DNA libraries was measured by Qubit dsDNA HS Assay (Thermo Fisher, United States, Catalog No. Q32851) and the quality was examined using the Agilent High Sensitivity DNA Kit (Agilent Technologies, United States, Catalog No. 5067-4626). High-throughput sequencing was performed on Illumina's X-Ten platform. The sequencing depth of each library was 80M and each library sequencing data was more than 25GB which were processed as previously reported [1]. Briefly, the sequencing quality was evaluated by the Illumina Sequencing Analysis Viewer and the FastQC software (Babraham Bioinformatics, Cambridge, UK). Sequencing adapters and 3’-low quality bases were trimmed from raw sequencing reads using a custom algorithm and then aligned to C->T in silico converted hg19 reference genome, using Bismark version 0.17.0 (Bowtie2 as the default aligner behind Bismark). Aligned reads were then evaluated by Picard version 2.5.0 for metrics that measured the performance of target-capture based bisulfite sequencing assays (<http://broadinstitute.github.io/picard>). The biases of specific motifs or GC enriched regions were excluded. After the preliminary analysis, we calculated the average coverage as well as the missing rate for each CpG site. The CpG sites with coverage less than 50X and/or with missing rate >0.20 were filtered out.

***De novel* marker discovery analysis**

Differentially methylated CpG sites were discovered using R package DSS version 2.14.0. Mann-Whitney U test was used to analyze methylation differences with mean β value difference greater than 0.2, a *p* value < 0.001, or a false discovery rate (FDR) < 0.05 among lymph node metastasis positive (LNM+) and negative (LNM-) samples. The hypermethylated and hypomethylated sites were identified. The differentially methylated regions (DMRs) based on the above differential methylation CpG sites was further obtained by co-methylation analysis [2, 3]. The co-methylation regions were annotated by 150 bp upstream and downstream of the differential methylation sites in GRCh37/hg19 reference genome, with a minimum of 4 CpG sites. A differential methylation region was defined by either a mean β value difference > 0.2 or log2 fold change of mean β value > 0.58, with a *p* value < 0.01 and FDR < 0.05. Targeted regions of methylation were selected as candidate features to build classification models of LNM positive /negative state. DMRs followed by technical validation using qPCR assay on the same frozen samples in the discovery cohort.

**Methylation analysis by qPCR**

The methylation analysis by MethyLight described earlier [3]. Briefly, the bisulfite-modified DNA was subjected to multiplex PCR amplification with specific primer designs for an enrichment of targeted regions. The reaction was carried out with thermal cycles as follow: 98°C for 30 sec; 20 cycles of 98°C for 15 sec, 60°C for 15 sec and 72°C for 15 sec; and 72°C for 5 min. The multiplex PCR products were further used for quantification of targeted regions of interest. The qPCR reaction was carried out with thermal cycles as follow: 95°C for 5 min; 40 cycles of 95°C for 15 sec, 62°C for 1 min and 62°C for acquiring fluorescent signal. Co-methylation levels of a genomic region of interest were expressed by ΔCt (cycle threshold), where ΔCt = Mean Ct (region of interest) - Mean Ct (region of control). The methylated bisulfite-converted DNA fragments of regions of interest were amplified by the assay. The ΔCT values were inversely correlated with the percentages of methylated molecules among total bisulfite-converted DNA molecules.

**Development and evaluation of the conventional prediction model, and the integrated model of 3-marker methylation and clinicopathological features**

The 8 clinicopathologic variables were included in the univariate analysis to explore the association with LNM in the model development cohort, and variables with a *p* value < 0.05 were included in multivariate analysis for the conventional model. Forward stepwise regression analysis evaluated odds ratio (OR) values with a 95% CI to identify independent predictors. The integrated model of methylation and clinicopathological features was built according to independent predictors and the 3-maker methylation model. Tolerance and variation inflation factors were used to evaluate the multicollinearity of multivariate models. Based on both multivariate logistic regression models, two quantitative scoring formulas were derived according to the coefficients and intercepts. The formula for the conventional model was logit (odds) = - 3.473 + 0.908 × SM + 1.349 × undifferentiated + 2.424 × LVI (presence) + 0.858 × ulceration (presence). The formula for the integrated model was logit (odds) = - 4.896 + 0.956 × SM + 1.351 × undifferentiated + 2.298 × LVI (presence) + 0.990 × ulceration (presence) + 2.869 × 3-marker methylation. The area under the receiver operating characteristic curve (ROC) and the corresponding area under the ROC curve (AUC values) was measured. The cutoff value of the model score is based on the maximum Youden index.

**Statistical analysis**

The Benjamini-Hochberg method was used to calibrate multiple hypothesis tests. R packages of ComplexHeatmap and Corrplot were used for unsupervised hierarchical clustering and correlation analysis, pROC (1.16.1) for ROC and AUC and AUC confidence interval calculations, ggplot2 (3.2.1) and RColorBrewer (1.1.2) for visualization of figures. Logistic regression-based model constructions were conducted using R glmnet (2.0.16) packages. The β-value and LNM risk score distribution between clinical categories were presented as box plots with median and the interquartile range marks. Differences between 2 groups were analyzed with the unpaired Student’s *t* test (2-tailed tests), and 1-way ANOVA followed by Dunnett’s multiple comparisons tests when more than 2 groups were compared. Pearson’s χ^2^ test was used to analyze the clinical variables on sensitivity and specificity. Pearson’s correlation analysis was performed to determine the correlation between 2 variables. Univariate and multivariate logistic regressions were used to evaluate the statistical significance of clinicopathological variables and the 3-marker methylation model. Comparison of AUC values were conducted by Hanley and McNeil tests or DeLong test, when appropriate. The sensitivity, specificity, and accuracy of 3-marker methylation model, conventional model, and integrated model in detecting LNM were obtained by comparison to pathology. A *p* value < 0.05 on two sides of all hypothesis tests were considered statistically significant. All statistical analysis and data visualization tools were from R (3.6.0) and GraphPad Prism 8.

**References**

1. Liang WH, Zhao Y, Huang WZ, Gao YB, Xu WH, Tao JS, et al. Non-invasive diagnosis of early-stage lung cancer using high-throughput targeted DNA methylation sequencing of circulating tumor DNA (ctDNA). Theranostics. 2019;9(7):2056–70.

2. Liang WH, Chen ZW, Li CC, Liu J, Tao JS, Liu X, et al. Accurate diagnosis of pulmonary nodules using a noninvasive DNA methylation test. J Clin Invest. 2021;131(10):e145973.

3. Li L, Ye ZJ, Yang S, Yang H, Jin J, Zhu YY, et al. Diagnosis of pulmonary nodules by DNA methylation analysis in bronchoalveolar lavage fluids. Clin Epigenetics. 2021;13(1):185.

**Table S1.** Clinical summary of EGC samples from genome-wide methylation sequencing in the marker discovery cohort

| **Characteristics** | **EGCs**  **n=47 (%)** |
| --- | --- |
| Gender |  |
| Male | 24 (51.1%) |
| Female | 23 (48.9%) |
| Age (years) |  |
| <60 | 27 (57.4%) |
| ≥60 | 20 (42.6%) |
| Tumor size (mm) |  |
| ≤20 | 20 (42.6%) |
| >20 | 27 (57.4%) |
| Invasional depth |  |
| M | 15 (31.9%) |
| SM | 32 (68.1%) |
| Differentiation |  |
| Differentiated | 18 (38.3%) |
| Undifferentiated | 29 (61.7%) |
| LVI |  |
| Presence | 18 (38.3%) |
| Absence | 29 (61.7%) |
| Ulceration |  |
| Presence | 16 (34.0%) |
| Absence | 31 (66.0%) |
| Tumor location |  |
| Upper | 4 (8.5%) |
| Middle | 6 (12.8%) |
| Lower | 37 (78.7%) |
| LNM |  |
| LNM+ | 23 (48.9%) |
| LNM- | 24 (51.1%) |

*M* mucosa, *SM* submucosa, *LNM* lymph node metastasis, *LVI* lymphovascular invasion, *LNM+* samples of EGC patients with positive lymph node metastasis, *LNM-* samples of EGC patients with negative lymph node metastasis

**Table S2**. Genomic information of the 50 methylation markers

| **Chromosome location (hg19)** | **Gene Name** | **Region sequences** |
| --- | --- | --- |
| chr20:  57429888-57429996 | GNAS | GACGCAAGATCCATCTCAGACCCCCCAGCCCCGAGATCCAGGCTGCCGATCCGCCTACTCCGCGGCCTACTCGCGCGTCTGCCTGGCGGGGCAAGTCCGAGAGCAGCCG |
| chr19:  40421516-40421618 | FCGBP | GTGCAGCCACACGACGAGGCCGGCACGCAGGCGCCGCCGCTGGCCACGAAGCCTGGGAGGCACACGCAGCCCTCCACGCAGGGGCGCCCGGAGCAGTTGGACG |
| chr8:  144790098-144790219 | CCDC166 | AAAGGCGTTCTCTCGCAGCACCTGGTCAACGCTCTCCTCGCAGGTGTCCAGCTGCTCCGAGAGCAGCGCGTGTTCGCGTTGCAGGTACTGCGCGCGCTCCGATAGCGGCTGCTCGGCACCCGC |
| chr7:  156720557-156720647 | LMBR1\|NOM1 | AGATGGAGTCTCGCTCTTGTCGCCTAGGCGGCCCCAATCCTGGGAGCGTGGTGCCGCGGGCCTGTCCATGCCTGATCTGTGCCTGGAGGCG |
| chr8:  145621816-145621943 | CPSF1 | TGAGTAGCCATAAATATCCTCGAAGTAGCGGAAACGCGCCACGCGGCCCCGGGCCCCAGCCCCCTCCTCTGCGCCGCCACCTTCTGCTTTCTTCTTGGATGGCTTTGGCTTCTTCACGGAAGTTGA |
| chr19:  46388274-46388361 | IRF2BP1_2 | CACCAGCCCGTGATCCTTCTTGAAGCGCACATTGAACGGGGCGCAGGCGGACAGCGCCAGTAGCTGTTCCCGCACTGCTTTGGGGCGC |
| chr5:  140799051-140799141 | PCDHGB7 | TCAGCGCCAATGTGAGCCTGCGCGTGTTGGTGGGCGACCGTAACGACAACGCACCGCGGGTGCTGTACCCTGCGCTGGGTCCCGACGGCTC |
| chr20:  54580328-54580455 | CBLN4 | GATCTGAGTCCGCAGAGGAGGCGGCTGGTCCCCTCACCCCTCCCAGCGCGGAACTCTGCCGCCGAGTGCCCTCCAAGCGGAGCGCGGCGCTGCCAGGCCGGGTCGCGAAAAAGGATAAGCCGCCGCGG |
| chr19:  46388150-46388257 | IRF2BP1_1 | GGCGAGCCACTGCCAGGACGCCGGCGTACACATTGCCGGAACCACAGGGGTATTCGGTGAAGAGCTTCAGCTCGAACTCGTATCCTGGAGGACGGGCAGTAGCATCGA |
| chr14:  48145215-48145317 | MDGA2\|MIR548Y | GGGCGAAGCGGCAAGCGCGGGGCGCAGGCGGACCGGCGGCCGGGCTGCTGGGCTGCTGGGCTGCCGGGGAGGAAGGCTGTGTGTGGTCACGTTGTGCGCTACG |
| chr12:  6729685-6729784 | LPAR5 | GGAAGATGACGAGGTTAGCCAGCAGGAGGCGCACGGTCTTCCGCCGCCGCTGGCTCTGCGTGGCGTCGGGGCGCGCCAGCGTCCAGAAGACTCGGCCCGA |
| chr19:  4054935-4055028 | ZBTB7A | ACGGTGAGCGTGGCCGTGTAGGCGAAGTCCATGAGCGCGGTGAGCGCCTCGGCGCTGACGAAGTCGATCTCGTACACGTTCTGCTGGTCCACCA |
| chr10:  15032901-15033003 | MEIG1\|OLAH | AATACCTTTGCCGTCTTCTGTACCAAGGCAGCCAAAAACACAGGCCCGTTGTCTGAGCCGATCCGTAAGGGCGGTCGAAATCTAGGAATCACATCTCGAAGAA |
| chr2:  115420295-115420419 | DPP10 | TTCCCGCATGGCGCTTTGGCTCCTGGGGTTCTGCTTGCAGCCCACAGGGGCCCTGAAGTGCCAGGGGGGGACCTTGGGCCACCAGGAGCGAGGCAGGCCACGATTTGAGGGCTTAGGTCCAGAGC |
| chr19:57351973-57352096 | PEG3 | CGGGCACGAACAGCCGCCTAGCGCACCCTCATGGCGCCCGGCGCCCGGCGGCGCCACCAGCCCAGGGTGGACATCTCCCGCGCCTCCCAAACCTCTCCTCCCGCAGCTGCCCAGACTTCTGCAC |
| chr2:  99439356-99439449 | KIAA1211L | GGGCTCAGGCGCCGGCCCTGGGGGCCCCTTCTCCTCATCCGGGAGCACGGGCGGCGTCGGCTCCGCTTCCTTCGGGACACTGCGTTCTGGCCCG |
| chr4:  20254755-20254857 | SLIT2 | GCGAGGACCGGTCCAGGCTGCGGCGGAGTCGAGGGCGAGGGAGAGGCCGCGTGAGTGAGCAGAGTCCAGAGCCGTGCGCCCCCAGAACTGCGCGTCCGCCCCG |
| chr13:  50707675-50707772 | DLEU1 | CCAGGGGCACGAAGGAACGGCGGCCGAGGGAAGTCCACGCGGGCTCTGGCGCAGCAGCGAAGCTGGAGGCGTTTGCTCCGCTCTGCCCGGAGCAAGGG |
| chr20:  59828347-59828472 | CDH4_2 | GGCGAGCGGGTCTCCCGCTGCCTCTGCAGAGCCCCGCGGGCCGGATTGCCAGCTTTGCTCTGCGCCTGGCGAGGTGCGCGGCCCGCGGGGGCAGAGAGCGCGGCGGCTCCGGGGGCGCCCCTGGGC |
| chr5:  3595940-3596055 | IRX1 | CAAATTGTGTCTGAAAGCCCCGCCGCCGAGCGGAGGGCGGCCGCCGCAGTCGGCGCGCGATTGCGGATCCGGGCGCAGCCGGGAGCCGGGCGCCTGCGAGCACCGGGCAGAGGAGC |
| chr17:  6617250-6617357 | SLC13A5 | ACGTCCGCCCCACTCTAGGTGCAGGACCCCTTTTCCCCGCTCGCACTCTCCGGCCCGGAGCTCCTGGGCGATCGCACAGGGAAGCGAGGCCACTGTCCTCCTCTGTCC |
| chr20:  43726583-43726711 | KCNS1 | TACTCGAGGCGTCGCAGCACCGGGTCGTCGCGCACGCCTTCCGGGCTGCGGCCCGCGGCCACCGCAGCCACGGCGGCCGCCGCCTCGCGGGCCTGGTACTCGGGCAGGCTGTGGATGCACATGGCGGCG |
| chr6:  85473356-85473484 | TBX18 | CAAACAGCCGCGGCCGAAGGCGCGGGTCGCCGAGTGGGCGGCGGCCGCCCGCGAGGCGAGCCTGGAAGCAGTATCTGAACCGTCTGGAGGGTGACCAGGTTGCGCGGCGAGCTTTGCGACTGACACGGC |
| chr7:  71801444-71801553 | CALN1 | GGCGGCCAGGAGCCAGCTCCACGGAAGGCAAGGTGGCCGGCGCGGCCCCCAGCCCGCGGGGTGGGTGCCCCAGTGTCGGAGCCAACAGGTACGGGAGGCGCTGTCTGCCA |
| chr8:  144790031-144790137 | CCDC166_3 | GCGCGCGCTCACGTAGCTGGCGTAGAGCCGGTTCTCCTCGCGCAGGCGCAGCGCCTCGCGGTCCAGGAAGGCGTTCTCTCGCAGCACCTGGTCAACGCTCTCCTCGC |
| chr20:  59827168-59827280 | CDH4_1 | GAGGCTGGGCTGGTGCGGCGCGGGGGGCGCGGGCGCGGGCTCTGCGCCTTTAAGGCCCGCGTCGGCAGAGCGAGCGCAGGGGCCGGGCGGCGGGGCGGGGCCTCGGCGGGGCG |
| chr10:  129535461-129535553 | FOXI2 | TGCTGGCGGCCAAGCTGGATGGGTCGCCAGTGAGTTTCGGTGCGGCACCGCTGGCCCAGGCCCGGGCGCGGCTGGACATGGCCACCTACTGCG |
| chr12:  107713264-107713358 | BTBD11 | CTGGCCGCACTGTCCCTCTACAACATGAGCAGCGCCGGCGGCGACCGCCTGGGCCGCGGCAAGTCGGCCCGCTGCGGCCTCACCTTCTCCGTGGG |
| chr8:  144789968-144790070 | CCDC166_2 | CTGCGCTAGGTCCACGCGGTTCTGCTCGTCCAGCCGGACGATGGCTTTGGCGCAGCGCTGGGCGCGCGCGCTCACGTAGCTGGCGTAGAGCCGGTTCTCCTCG |
| chr12:  52644237-52644318 | KRT7 | CAAAGTGGGGCGCTCAGGCGAAGCGCACGGAGCGGCCGCTGCCGCTGCTCTGGCCGCAGGGCGCACACAGGCCGGTGCTCAC |
| chr19:  46388013-46388129 | IRF2BP1_3 | GCTGCGGACGCCGTCGGTAAGCAGCTCGCCCAGCTGCCGCCATTCTCCTGATCCATGCCGGCGTTCATATTCGAGGTACTTGAAGCCCGAAGAAGCCAGTGCCTTGCCCGGCTCCCG |
| chr13:  113765063-113765189 | F7 | TTCCTGGAGGAGCTGCGGCCGGGCTCCCTGGAGAGGGAGTGCAAGGAGGAGCAGTGCTCCTTCGAGGAGGCCCGGGAGATCTTCAAGGACGCGGAGAGGACGGTGAGCCCAGCCTCGGGGCGCCCCG |
| chr20:  61638511-61638635 | BHLHE23 | CCTGGAGCGCTAGTACTTGCTTCTCGACTCCCCGGCCGCCGCCTCCGGCCGCCCCGGGGATTCCGCCACCAAACGCACGCGTCCCAGGTGGGCACCCGCCTCGGTCCGTCTTTGAGTCTGACCCT |
| chr20:  61472086-61472178 | COL9A3 | ATTCAACGTGAGGAAGCAAGTGACAAGGACGCCCGAAGCACAGTGGACGGTCATGAAGGAGCGGGGGTGTGGCAGGCGGGTGACGTCCAGGAG |
| chr16:  55358624-55358747 | IRX6 | GGATTACCGTGACGTCACATTGAGCCTCTGGCCACCTTGGACTGGGACACCTCCGGAGCCTCACAGCCCCGCGCCGCGCCGCGCCTCACCTCGCCACCACGCGCCTTTGGGAACCCGCATCTTC |
| chr16:  22825695-22825782 | HS3ST2 | GGACCGGGAGATGCTGGAAATGCAACCGCCTGTTCCCCGAGGAGCCGCTGCCCCCGGGACCCCCTGGCACTGTGCGCACCCTGGTCAG |
| chr5:  2749119-2749218 | IRX2 | CGGCCGAGCACGAGTGATCCGTGAGCGAGTCCACGTGCAGGCTGATCCCTGTGGGGGCGCGGGCACGGTGGGTGGCACGAGGGGACAGCGCAGGCACCTG |
| chr19:  12876971-12877068 | HOOK2 | TGCAGCTCCTCCTGCCGCTCCCGGTCGGCCGCCTCCTGCCTGCACAGCCGCTTGTTCTCCAGCTGAAGCCGCAGGAGCGTCTCCCTGCAGACCCGGGA |
| chr7:  98971435-98971547 | ARPC1B | TTCACCGTGATAGCCAGGATGGTCTCGATCTGAGCTCGTGATCCTCCCGCCTCGGCCTCCCAAAGTGCTGAGATTACAGGCGTGAGCCACCGCGACCGGCCGAGGTAAGGTTT |
| chr7:  47092560-47092685 | IGFBP3\|TNS3 | TCGCCTTCGTCCGGTTTCACTACCGGAGCGACGCGCAAGACGCCGAGGCCGCCATGGACTGGGCGGTGCTGGACGGACGCGAGCTGCGGGTGCAGATGGCGCGCTATGGCCGCCGGGACCTGCCTC |
| chr16:  86542248-86542341 | FENDRR | GAGCAGCACTCACCTGGGTCTGTGCGCGCCGCGATGCGCCGGGGCTCCTGCGCTGTCCTGCGCTCCGCTCTCGGCACCACCTTTCGTGGCTCCA |
| chr19:  55593523-55593602 | EPS8L1 | AGATCAAGTACGCCTTCAGCCTGCTGGTGAGGACGCGCCCGCCCCTGGGCCGGGGCGCGGGCACGACGAACCTGTCCCGT |
| chr2:  210636431-210636549 | UNC80 | GTACCCCTGCGGCTCAAGCTGCCCCGGATTCGCTTCTCCGGCCGTGCAGCCCGGCGCGGGCCGCTGTCCACAGTGGGAGGTGCTGAAAGCAGGGAGCGGGTGCGGGGGCGGCGAGGCGG |
| chr5:  163754180-163754304 | MAT2B\|LOC101927835 | CAAGGCCAGGGCGAAATTAGAATTACTGATGAGGTTCCATGTCCCACGGGGCACGCATTTTCATTGATAAACATCTTAACAGGAAACAGGGTTTGAGAGCAGACAATCAGTCTGACTAGAATTCG |
| chr1:  53387805-53387929 | ECHDC2 | GCTGTGTCGCCCAGGTTGGAGTGCTGTGCCTTGATCTTGGCTCACTGTAACCTCCGCCTCCGGGTTCAAGCGATTCTCCTGCCTCAACCTCCCAAGTAGCTGGGATTACAGGGACGCGCCACCAC |
| chr12:  42873109-42873200 | PRICKLE1 | CCAAGTTATTGACCAACGTTTCCAGCTGCTTCGGTAGCAGCTTCTAACCAGTCCCCACATAAAGCCTCCAATACCATACACATAGCGTGTGT |
| chr6:  118228603-118228696 | SLC35F1 | GGGCGGGCCAGGACTTGGGGACGCGGCTCGGGAAGAGCCGGGGCGGGCGGCGGCGGCGGCGGCACGGGCGCGAGGGTGCGCGCACTGGGACTGG |
| chr8:  81789920-81790033 | ZNF704\|PAG1 | CGTTCATTCTGCATGGATTTGTTGAGTCCTGACAAGTGCCAGGTCTTGTTCTTGGTGCTGGCGTGCAGCAGCGAACAAAATAACCCTCCCTCGGGGAGGAAGCAGACAATAATC |
| chr21:  22370424-22370510 | NCAM2 | CGGCGGGCGAGTGGCGCCTTCGCGATCGCGGCCGCCCCTCTAGGCGGAGGCACCGGGGACCGAACCCGTGACTTCGAGCTCCACTGC |
| chr8:  70602364-70602463 | SLCO5A1 | ACAGGGTCTTCTTTCCCCGCTGATTCCGCCAAGCCCGTTCCCTTGGCTGTGGTTTCGCTGGATAGTAGGTAGGGACAGTGGGAATCTCGTTCATCCATTC |

Different regions of the same gene are annotated as gene_number. An intergenic region is annotated as gene1/gene2, where gene 1 and gene 2 are the two adjacent genes of the intergenic region.

**Table S3.** The conventional prediction model for identification of LNM

| **Characteristics** | **β** | **OR (95% CI)** | ***p* value** |
| --- | --- | --- | --- |
| Invasional depth (SM vs. M) | 0.908 | 2.48 (1.41-4.36) | 0.002 |
| Differentiation (Undifferentiated vs. Differentiated) | 1.349 | 3.85 (1.94-7.67) | < 0.001 |
| LVI (Presence vs. Absence) | 2.424 | 11.30 (5.40-23.64) | < 0.001 |
| Ulceration (Presence vs. Absence) | 0.858 | 2.36 (1.39-4.00) | 0.001 |
| Constant | -3.473 | 0.03 | < 0.001 |

*OR* odds ratio, *CI* confidence interval, *M* mucosa, *SM* submucosa, *LVI* lymphovascular invasion

**Table S4.** The integrated model for identification of LNM

| **Characteristics** | **β** | **OR (95% CI)** | ***p* value** |
| --- | --- | --- | --- |
| Invasional depth (SM vs. M) | 0.956 | 2.60 (1.35-5.04) | 0.005 |
| Differentiation (Undifferentiated vs. Differentiated) | 1.351 | 3.86 (1.73-8.61) | 0.001 |
| LVI (Presence vs. Absence) | 2.298 | 9.96 (4.14-23.92) | < 0.001 |
| Ulceration (Presence vs. Absence) | 0.990 | 2.69 (1.44-5.02) | 0.002 |
| 3-gene methylation (Risk score) | 2.869 | 17.62 (9.14-33.94) | < 0.001 |
| Constant | -4.986 | 0.01 | < 0.001 |

*OR* odds ratio, *CI* confidence interval, *M* mucosa, *SM* submucosa, *LVI* lymphovascular invasion

**Table S5.** Multicollinearity assessment in the conventional prediction model

| **Predictors** | **Tolerance** | **Variance Inflation Factor** |
| --- | --- | --- |
| Invasional depth | 0.926 | 1.080 |
| Differentiation | 0.417 | 2.397 |
| LVI | 0.924 | 1.082 |
| Ulceration | 0.977 | 1.024 |

*LVI* lymphovascular invasion

**Table S6.** Multicollinearity assessment in the integrated model

| **Predictors** | **Tolerance** | **Variance Inflation Factor** |
| --- | --- | --- |
| Invasional depth | 0.924 | 1.083 |
| LVI | 0.907 | 1.102 |
| Ulceration | 0.981 | 1.019 |
| Differentiation | 0.959 | 1.043 |
| 3-marker methylation | 0.941 | 1.062 |

*LVI* lymphovascular invasion
